# The Efficacy and Safety of Bisphosphonate Therapy for Osteopenia/Osteoporosis in Patients With Chronic Kidney Disease: A Systematic Review and Individual Patient-Level Meta-Analysis of Placebo-Controlled Randomized Trials

**DOI:** 10.1177/20543581241283523

**Published:** 2024-10-08

**Authors:** Reid Whitlock, Kerry MacDonald, Navdeep Tangri, Michael Walsh, David Collister

**Affiliations:** 1 Chronic Disease Innovation Centre, Winnipeg, MB, Canada; 2 University of Manitoba Libraries, Winnipeg, MB, Canada; 3 Section of Nephrology, Department of Medicine, University of Manitoba, Winnipeg, MB, Canada; 4 Division of Nephrology, Department of Medicine, McMaster University, Hamilton, ON, Canada; 5 Population Health Research Institute, Hamilton, ON, Canada; 6 Division of Nephrology, Department of Medicine, Faculty of Medicine & Dentistry, University of Alberta, Edmonton, AB Canada

**Keywords:** bisphosphonates, chronic kidney disease, individual patient-level data

## Abstract

**Background:**

The efficacy and safety of bisphosphonate therapy for the treatment of osteoporosis and osteopenia in the setting of chronic kidney disease (CKD) is unclear.

**Objective:**

To determine the effect of bisphosphonate therapy on fractures, bone mineral density (BMD), and adverse events in adults across the spectrum of CKD and dialysis.

**Design:**

Systematic review and individual patient-level meta-analysis.

**Setting:**

Searches of Ageline, CINAHL, the Cochrane Library, EMBASE, and Medline from inception to August 25, 2016, supplemented with manual screening and clinicalstudydatarequest.com. Authors were contacted for individual patient-level data.

**Patients:**

Randomized, placebo-controlled trials with 100 or more participants that evaluated the treatment of primary osteoporosis/osteopenia in adult men and women with bisphosphonate therapy.

**Measurements:**

Study characteristics, quality, and data were assessed independently by 2 reviewers. Outcome measures were fractures, BMD, and adverse events including decline in estimated glomerular filtration rate (eGFR) and hypocalcemia (calcium <2.00 mmol/L).

**Methods:**

Single-stage individual patient-level meta-analysis.

**Results:**

Of 39 eligible studies, individual patient-level data was available for 7 studies, all of which were studies of ibandronate. Of 7428 participants (5010 ibandronate, 2418 placebo), 100% were female, 98.6% were white, the mean body mass index was 25.7 kg/m^2^ (SD 3.9), 18.9% were smokers and there were 740 fracture events. The mean eGFR was 69.1 mL/min/1.73 m^2^ (SD 15.9) including 14.5%, 54.9%, 27.5%, 3.0%, and 0.2% stages G1, G2, G3A, G3B, and G4 CKD. Ibandronate increased hip and lumbar spine BMD and decreased the risk of fracture in the overall population (hazard ratio (HR) 0.871, 95% confidence interval (CI) 0.746, 1.018) but in patients with stage G3B CKD, it increased the risk of fracture (HR 3.862, 95% CI 1.156, 12.903). Ibandronate did not impact eGFR over 12 months but increased the risk of hypocalcemia (HR 1.324, 95% CI 1.056, 1.660) with no evidence of any effect modification by CKD stage (all tests of interaction *p* > 0.05).

**Limitations:**

Clinically significant heterogeneity among studies, lack of long-term follow-up and bone biopsy results, limited representation of stage G4 and G5 CKD patients.

**Conclusions:**

Chronic kidney disease potentially modifies the efficacy but not the safety of bisphosphonate therapy in osteopenia and osteoporosis.

**Registration:**

PROSPERO CRD42020145613

## Abrégé

**Contexte:**

L’efficacité et l’innocuité du traitement de l’ostéoporose et de l’ostéopénie par les bisphosphonates en contexte d’insuffisance rénale chronique (IRC) sont mal connues.

**Objectif:**

Déterminer l’effet du traitement par bisphosphonates sur les fractures, la densité minérale osseuse (DMO) et les événements indésirables chez les adultes dans tout le spectre de l’IRC et de la dialyse.

**Conception:**

Revue systématique et méta-analyse individuelle au niveau du patient.

**Cadre:**

Recherches sur Ageline, CINAHL, la bibliothèque Cochrane, EMBASE et Medline depuis leur création jusqu’au 25 août 2016, complétées par une sélection manuelle et la consultation de clinicalstudydatarequest.com. Les auteurs ont été contactés pour l’obtention des données individuelles des patients.

**Sujets:**

Les essais randomisés contrôlés contre placebo qui ont été menés auprès d’au moins 100 sujets et qui ont évalué le traitement de l’ostéoporose/ostéopénie primaire chez des hommes et femmes adultes prenant des bisphosphonates.

**Mesures:**

Les caractéristiques, la qualité et les données de l’étude ont été examinées de manière indépendante par deux évaluateurs. Les critères d’évaluation étaient les fractures, la DMO et les événements indésirables, y compris la réduction du débit de filtration glomérulaire estimé (DFGe) et l’hypocalcémie (calcium < 2,00 mmol/L).

**Méthodologie:**

Méta-analyse en une seule étape au niveau du patient.

**Résultats:**

Sur les 39 études admissibles, seules 7 études présentaient des données individuelles au niveau des patients et celles-ci portaient toutes sur l’ibandronate. Les 7428 sujets (5010 ibandronate, 2418 placebo) inclus étaient des femmes (98,6 % étaient de race blanche) avec un indice de masse corporelle moyen de 25,7 kg/m^2^ (écart-type : 3,9), 18,9 % étaient fumeuses et on comptait 740 fractures. Le DFGe moyen était de 69,1 ml/min/1,73 m^2^ (écart-type : 15,9) pour des sujets atteints d’IRC de stades G1 (14,5 %), G2 (54,9 %), G3 (27,5 %), G3B (3,0 %) et G4 (0,2 %). L’ibandronate a amélioré la DMO de la hanche et du rachis lombaire et diminué le risque de fracture dans l’ensemble de la population (RR : 0,871; IC 95 % : 0,746-1,018), mais a augmenté le risque de fractures chez les sujets atteints d’IRC de stade G3B (RR : 3,862; IC95 % : 1,156-12,903). Sur une période de 12 mois, le DFGe n’a pas semblé affecté par le traitement à l’ibandronate, mais on a observé une augmentation du risque d’hypocalcémie (RR : 1,324; IC95 % : 1,056-1,660) sans modification de l’effet selon le stade de l’IRC (*p*>0,05 pour tous les tests d’interaction).

**Limites:**

Hétérogénéité cliniquement significative entre les études; absence de suivi à long terme et de résultats de biopsies osseuses; représentation limitée des patients atteints d’IRC de stade G4 et G5.

**Conclusion:**

L’IRC peut modifier l’efficacité, mais non l’innocuité, des bisphosphonates pour le traitement de l’ostéopénie et de l’ostéoporose.

## Introduction

Chronic kidney disease (CKD) is associated with an increased risk of fracture, and fracture-related morbidity, mortality, and health resource utilization due to CKD-related mineral bone disorder (MBD).^1-3^ However, treatments for MBD that reduce fractures are limited.^4^ Osteopenia and osteoporosis are common in the general population and increase the risk of vertebral and non-vertebral (hip, radius, other) fractures and their associated morbidity and mortality.^5-7^ Aging is a shared risk factor for both CKD and decreased bone mineral density (BMD), so CKD and osteopenia/osteoporosis frequently co-exist.

Bisphosphonates increase BMD across skeletal sites and decrease vertebral and non-vertebral fractures, but their efficacy and safety in the setting of CKD are uncertain.^8^ Bisphosphonates are associated with adverse events including kidney^9^ (acute tubular necrosis, collapsing focal segmental glomerulosclerosis) and non-kidney^10-12^ (osteonecrosis, atypical fractures, hypocalcemia, arrhythmias) complications which may be modified by CKD given their excretion by the kidneys^13^ and the coexistence of CKD-MBD.

Our previous work has shown that measures of BMD and risk scores that incorporate BMD (Fracture Risk Assessment Tool (FRAX)) discriminate equally well in individuals with or without CKD.^14^ In routine clinical practice, these scores are used to identify patients at high risk of fractures and recommend treatment with bisphosphonates. However, little is known about the efficacy and safety of bisphosphonates in patients across the spectrum of CKD. We performed a systematic review and individual patient-level meta-analysis of randomized placebo-controlled trials of bisphosphonates for the treatment of osteopenia/osteoporosis in adults to determine if the efficacy and safety of bisphosphonates is modified by CKD.

## Methods

### Data Sources and Searches

We developed and followed a protocol that included PICO (population, intervention, comparison, outcomes) criteria that was registered at PROSPERO.^15^ We followed the Preferred Reporting Items for Systematic Reviews and Meta-Analyses guidelines for reporting of systematic reviews and meta-analyses.^16^

Our initial search strategy focused on CKD and kidney failure (hemodialysis, peritoneal dialysis but not kidney transplantation), but given the paucity of studies, we removed our kidney disease filter and included general population studies with individual patient-level data to identify CKD subgroups. We identified randomized placebo-controlled trials of bisphosphonate therapy for the treatment of primary osteopenia (T-score −1.0 to −2.5 SD) and osteoporosis (T-score −2.5 SD or below, history of fragility fracture) in adult men or women. The studies had to report vertebral or non-vertebral fractures (hip, radius, other), BMD measurements by dual-energy X-ray absorptiometry (DEXA), or other imaging or adverse events in follow-up. We included studies with 100 or more participants and limited by English language and at least 1 year of follow-up. Studies focusing on secondary forms of osteopenia or osteoporosis other than hypogonadism were excluded (eg, asthma, sarcoidosis, anorexia nervosa, celiac disease, primary biliary sclerosis, multiple sclerosis, ankylosing spondylitis, neuromuscular diseases, and Cushing’s syndrome) in addition to those with other disorders affecting bone metabolism (eg, Paget’s disease, malignancy, and primary hyperparathyroidism).

We placed no limits on type of bisphosphonate (oral vs intravenous), dosing, schedule (continuous vs intermittent), and permitted calcium and vitamin D supplementation and any other co-intervention known to affect bone metabolism. We also excluded studies that included previous treatment with bisphosphonates or extension of bisphosphonate therapy. In collaboration with a medical librarian (KM), we retrieved information for the study from the following databases: Ageline, CINAHL, the Cochrane Library, EMBASE, and Medline. Our search of these databases ranged from the date of their establishment until August 25, 2016.

The search strategy was tailored to each database and used a combination of key terms, such as osteopenia, osteoporosis, and bisphosphonate. Medical Subject Heading terms were applied in the search strategy (see Supplemental Figure 1). We downloaded all of the received citations into RefWorks, version 2.0 (RefWorks-COS, Bethesda, Maryland). Two reviewers (DC and RW) independently reviewed each citation by title and abstract, and articles were selected for full-text review. Two reviewers (DC and RW) screened the reference lists of articles selected for full-text review. Full-text articles were finalized for inclusion after consultation with a third reviewer (NT). All disagreements were resolved by consensus.

We contacted corresponding authors via email of all included studies to request individual patient data to identify CKD subgroups. After 2 weeks, a second request was sent and only the authors of the studies who responded were included in the meta-analysis. Due to a lack of response by authors, we also contacted sponsors for data in a similar fashion. We also registered the study on clinicalstudydatarequest.com to obtain individual patient-level data from sponsors.

### Data Extraction and Quality Assessment

We created a data extraction form to capture relevant information from the included studies. For each eligible study, 2 reviewers (DC and RW) independently abstracted the relevant data; inconsistencies were corrected and resolved by consensus and consultation with a third reviewer (NT). Specifically, they abstracted study year, sample size, intervention, duration, co-interventions, assessments, and the primary outcome. Two reviewers (DC and RW) assessed studies for their quality of reporting and risk of bias using the Cochrane Collaboration’s tool for assessing risk of bias.^17^ Categories included random sequence generation, allocation concealment, blinding of participants and personnel, blinding of outcome assessment, incomplete outcome data, and selective reporting. Conflict was resolved by a third reviewer (NT).

### Data Synthesis and Analysis

The primary outcome was the composite of vertebral and non-vertebral fractures between bisphosphonate and placebo groups. Secondary outcomes included the changes in total hip BMD and total spine BMD at 1 year, the change in serum creatinine and proteinuria over 1 year and the time to hypocalcemia (calcium<2.00 mmol/L). We did not examine other safety outcomes given their rare incidence and the short duration of follow-up in most studies. Time to event outcomes were analyzed in shared frailty Cox proportional hazards (PH) models clustered by study that included treatment with bisphosphonate (pooled doses or administration schedules if applicable) or placebo and stratified by CKD subgroups (stage G1, G2, G3A, G3B, G4A, and G5 with estimated glomerular filtration rate (eGFR) calculated by the CKD-EPI equation) or eGFR modeled continuously with a treatment x CKD/eGFR interaction term. Violations of the Cox PH assumption were tested for using treatment-by-time interactions. Continuous outcomes (BMD and eGFR) were analyzed in mixed linear regression models using an unstructured covariance matrix with the same approach but adjusted for baseline values. Models were not adjusted for other covariates given randomization and the number of participants with presumably a balance of prognostic variables. Individual patient-level meta-analysis was performed using a single-stage approach with study and intercept as random effects and treatment as a fixed effect. Participant and time were modeled as random effects in the case or repeated measures. We did not perform any subgroup analyses. All statistical analyses were performed by using the SAS software, version 9.3 (SAS Institute, Inc.).

## Results

### Included Studies

**Figure 1.**
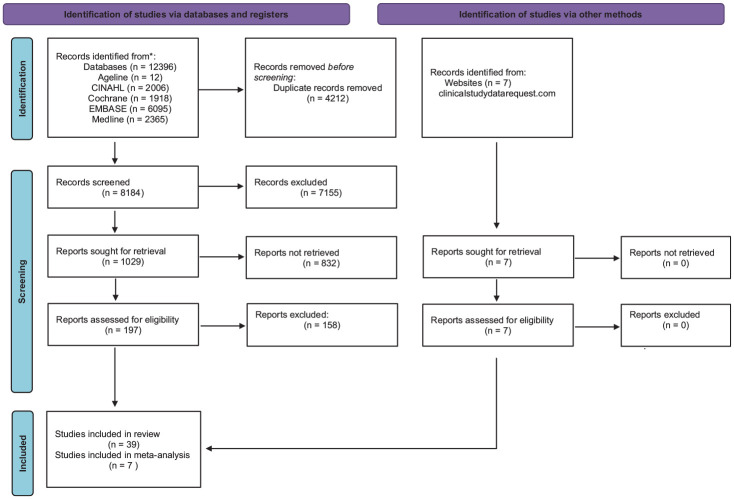
PRISMA flow diagram.

The PRISMA flow diagram of included studies is shown in Figure 1. Our search strategy retrieved 12396 studies, including 8184 unique studies for title screening. Of these, 1029 were selected for abstract screening, 197 were selected for full-text review, and 39 met the criteria for inclusion in our systematic review (Supplemental Table 1). Of the studies that met inclusion criteria, 7 studies provided individual patient-level data for meta-analysis, all from clinicalstudydatarequest.com (Supplemental Table 2).

### Quality of Reporting and Risk of Bias

The 7 studies that provided individual patient-level data for meta-analysis were reviewed for risk of bias (Supplemental Figure 2). One study was rated as a low risk of bias in all 7 domains, 1 was rated as a low risk of bias in 6 of 7 domains, and the remaining 5 studies were rated as a low risk of bias in 4 domains or fewer. Selective outcome reporting was the most common reason for high risk of bias, and a lack of description of allocation concealment was the most common reason for unclear risk of bias.

### Characteristics of Included Studies and Participants

Of the 7 included studies, all evaluated ibandronate versus placebo. No placebo-controlled trial of alendronate, risedronate, minodronate, or zoledronic acid provided individual patient-level data. Participant characteristics are shown in Table 1. A total of 7450 were included in the 7 studies. We excluded 22 who did not have a serum creatinine measured at baseline. Of 7428 participants included for analysis, 5010 were treated with ibandronate and 2418 were treated with placebo. Notably, 100% were female, 98.6% were white, 0.3% were black, 0.6% were Asian, and 0.5% were another race. The mean age was 66.2 (SD 6.8) years, the mean body mass index was 25.7 kg/m^2^ (SD 3.9), 18.9% were smokers, 0.74% had a history of fracture, 0.4% had a family history of fracture, 0.2% received corticosteroids, and 1.6% had rheumatoid arthritis. Study duration ranged from 1 year to 3 years.

**Table 1.** Characteristics of Included Participants.

		*N* (%)	Missing	Mean	SD	Median	IQR
	Age (years)	7428	0	66.2	6.8	67.0	61.5, 71.0
	Female sex	7428 (100%)	0		100.0		
Race	White	7322 (98.6%)	0				
	Black	24 (0.3%)	0				
	Asian	43 (0.6%)	0				
	Other	39 (0.5%)	0				
	Body mass index	7420	8	25.7	3.9	25.3	23.0, 28.0
	Total hip BMD (g/cm^2^)	2973	4455	0.779	0.12	0.773	0.695, 0.857
	Total spine BMD (g/cm^2^)	3101	4327	0.805	0.12	0.801	0.723, 0.881
	eGFR (mL/min/1.73 m^2^)	7428	0	69.1	15.9	66	57, 78
CKD stage	G1 (eGFR >90 mL/min/1.73 m^2^)	1075 (14.5%)	0				
	G2 (eGFR 60-89 mL/min/1.73 m^2^)	4078 (54.9%)	0				
	G3A (eGFR 45-59 mL/min/1.73 m^2^)	2043 (27.5%)	0				
	G3B (eGFR 30-44 mL/min/1.73 m^2^)	220 (3.0%)	0				
	G4 (eGFR 15-29 mL/min/1.73 m^2^)	12 (0.2%)	0				
	G5 (eGFR <15 mL/min/1.73 m^2^)	0 (0%)	0				
	Urine albumin/creatinine (mg/g)	518	6910	14.1	24.7	7	4, 15
	Urine protein/creatinine (mg/g)	355	7073	61.3	61.3	47	34, 70
	History of fracture	553 (7.4%)	0				
	Smoking	1406 (18.9%)	361				
	Family history of fracture	29 (0.4%)	7268				
	Corticosteroids	18 (0.2%)	0				
	Rheumatoid arthritis	116 (1.6%)	94				
	Parathyroid hormone (pmol/L)	499	6929	4.3	1.9	3.9	3.1, 5
	Serum calcium (mmol/L)	6869	559	2.40	0.10	2.40	2.35, 2.47
	Phosphate (mmol/L)	7188	240	1.18	0.15	1.16	1.07, 1.26
	Alkaline phosphatase (U/L)	7268	160	90.1	43.3	78	63, 103
	Bone-specific alkaline phosphatase (U/L)	1950	5478	73.6	54.5	53	37, 89
	Bisphosphonate	5010	0				
	Placebo	2418	0				

The mean eGFR was 69.1 mL/min/1.73 m^2^ (SD 15.9) including 14.5% with stage G1 (eGFR ≥ 90 mL/min/1.73 m^2^), 54.9% with stage G2 (eGFR 60-89 mL/min/1.73 m^2^), 27.5% with stage G3A (eGFR 45-59 mL/min/1.73 m^2^), 3.0% with stage G3B (eGFR 30-44 mL/min/1.73 m^2^) and 0.2% stage G4 (eGFR 15-29 mL/min/1.73 m^2^). There were no participants with stage G5 or G5D with kidney failure treated with dialysis. Urine ACR and PCR were collected in 518 and 355 participants with median (interquartile range (IQR)) of 7 mg/g (4, 15) and 47 mg/g (34, 70), respectively. The mean baseline (SD) total hip BMD was 0.779 g/cm^2^ (0.12) and the mean (SD) baseline total spine BMD was 0.805 g/cm^2^ (0.12). The mean (SD) baseline PTH was 4.3 pmol/L (1.9), serum calcium was 2.40 mmol/L (0.10), phosphate was 1.18 mmol/L (0.15), alkaline phosphatase was 160 U/L (90.1), and bone specific-alkaline phosphatase was 73.6 U/L (54.5).

### Fractures

The mean follow-up time was 2.1 ± 1.0 years and there were a total of 740 fractures in 7428 individuals. In Cox PH models, bisphosphonate therapy may decrease the risk of fracture (HR 0.871, 95% CI 0.746, 1.018), but this was not significantly different across CKD subgroups (G1 HR 0.797, 95% CI 0.427, 1.489, G2 0.851, 95% CI 0.700, 1.035, G3A HR 0.858, 95% CI 0.658, 1.119, tests of interaction all *p* > .05) except for G3B where bisphosphonate therapy increased the risk of fracture (HR 3.862, 95% CI 1.156, 12.903, test of interaction *p* < .05, see Table 2 and Supplemental Table 4). Chronic kidney disease stage (G2, G3A, G3B vs G1) and eGFR were not associated with fracture in both participants receiving bisphosphonate therapy as well as those treated with placebo (Table 2). We were unable to calculate effect sizes for the G4 subgroup given the limited number of participants. Given the limited number of fractures by site, we did not perform separate analyses by hip, vertebral, radius, or sites. Given the limited number of atypical fractures/osteonecrosis and differential classification across trials, we did not analyze this outcome.

**Table 2.** Cox PH Model for Fractures.

	Treatment	CKD	Hazard ratio	95% CI	
CKD models	Placebo	Stage G1 CKD (eGFR >90 mL/min/1.73 m^2^) (reference)			
		Stage G2 CKD (eGFR 60-89 mL/min/1.73 m^2^)	1.156	0.666	2.008
		Stage G3A CKD (eGFR 45-59 mL/min/1.73 m^2^)	1.095	0.618	1.943
		Stage G3B CKD (eGFR 30-44 mL/min/1.73 m^2^)	0.368	0.105	1.286
		Stage G4 CKD (eGFR 15-29 mL/min/1.73 m^2^)	n/a	n/a	n/a
	Bisphosphonate	Stage G1 CKD (eGFR >90 mL/min/1.73 m^2^) (reference)			
		Stage G2 CKD (eGFR 60-89 mL/min/1.73 m^2^)	1.235	0.815	1.870
		Stage G3A CKD (eGFR 45-59 mL/min/1.73 m^2^)	1.180	0.764	1.821
		Stage G3B CKD (eGFR 30-44 mL/min/1.73 m^2^)	1.784	0.998	3.189
		Stage G4 CKD (eGFR 15-29 mL/min/1.73 m^2^)	n/a	n/a	n/a
	Bisphosphonate versus Placebo	Stage G1 CKD (eGFR > 90 mL/min/1.73 m^2^)	0.797	0.427	1.489
		Stage G2 CKD (eGFR 60-89 mL/min/1.73 m^2^)	0.851	0.700	1.035
		Stage G3A CKD (eGFR 45-59 mL/min/1.73 m^2^)	0.858	0.658	1.119
		Stage G3B CKD (eGFR 30-44 mL/min/1.73 m^2^)	**3.862**	**1.156**	**12.903**
		Stage G4 CKD (eGFR 15-29 mL/min/1.73 m^2^)	n/a	n/a	n/a
		Placebo (reference)			
		Bisphosphonate^a^	0.884	0.760	1.027
eGFR models	Placebo	eGFR	1.005	0.996	1.015
	Bisphosphonate	eGFR	1.001	0.994	1.008
	Bisphosphonate versus Placebo	Placebo (reference)			
		Bisphosphonate^b^	0.871	0.746	1.018

*Note*. CKD = chronic kidney disease; CI = confidence interval. Bold values signify *p* < 0 .05.

a At all stages of CKD.

b At mean eGFR 69 mL/min/1.73 m^2^, 740 events.

### Bone Mineral Density

The impact of bisphosphonate therapy versus placebo on hip and lumbar spine BMD at 12 months is shown in Tables 3 and 4. There were a total of 2488 individuals with a hip BMD and 2617 individuals with a lumbar spine BMD at baseline and 12 months. Compared with placebo, bisphosphonate therapy increased BMD at both the hip (β 0.01493, SE 0.00166, *p* < .0001) and lumbar spine (β 0.02451, SE 0.002533, *p* < .0001). There was no difference in efficacy among CKD subgroups for hip BMD (tests of interaction *p* > .05), but there was increased efficacy for lumbar spine BMD in the Stage G4 CKD subgroup (β 0.2043, SE 0.04468, *p* < .0001) but whether this translated into improved clinical outcomes in uncertain as we were unable to calculate fracture risk in this subgroup.

**Table 3.** Linear Regression Model for Hip Bone Mineral Density.

		β	SE	*P* value
CKD model	Placebo (reference)			
	Bisphosphonate	**0.01493**	**0.001660**	**<.0001**
	Stage G1 CKD (eGFR >90 mL/min/1.73 m^2^) (reference)			
	Stage G2 CKD (eGFR 60-89 mL/min/1.73 m^2^)	0.001615	0.001837	.3793
	Stage G3A CKD (eGFR 45-59 mL/min/1.73 m^2^)	0.000080	0.002356	.9728
	Stage G3B CKD (eGFR 30-44 mL/min/1.73 m^2^)	0.002166	0.007344	.7681
	Stage G4 CKD (eGFR 15-29 mL/min/1.73 m^2^)	−0.00584	0.02030	.7738
	Bisphosphonate * Stage G2 CKD (eGFR 60-89 mL/min/1.73 m^2^)	−0.00049	0.002051	.8097
	Bisphosphonate * Stage G3A CKD (eGFR 45-59 mL/min/1.73 m^2^)	0.001415	0.002634	.5913
	Bisphosphonate * Stage G3B CKD (eGFR 30-44 mL/min/1.73 m^2^)	−0.00112	0.008345	.8933
	Bisphosphonate * Stage G4 CKD (eGFR 15-29 mL/min/1.73 m^2^)	−0.03068	0.02868	.2849
eGFR model	Placebo (reference)			
	Bisphosphonate	**0.01328**	**0.003963**	**.0008**
	eGFR	−0.00002	0.000048	.6115
	Bisphosphonate * eGFR	0.000022	0.000051	.6671

*Note*. CKD = chronic kidney disease; eGFR=estimated glomerular filtration rate; SE = standard error.

*N* = 2488.

Bold values signify *p* < 0.05.

**Table 4.** Linear Regression Model for Lumbar Spine Bone Mineral Density.

		β	SE	*P* value
CKD model	Placebo (reference)			
	Bisphosphonate	**0.02451**	**0.002533**	**<.0001**
	Stage G1 CKD (eGFR >90 mL/min/1.73 m^2^) (reference)			
	Stage G2 CKD (eGFR 60-89 mL/min/1.73 m^2^)	−0.00155	0.002789	.5782
	Stage G3A CKD (eGFR 45-59 mL/min/1.73 m^2^)	−0.00119	0.003583	.7399
	Stage G3B CKD (eGFR 30-44 mL/min/1.73 m^2^)	**0.02707**	**0.009829**	**.0059**
	Stage G4 CKD (eGFR 15-29 mL/min/1.73 m^2^)	**–0.07261**	**0.03162**	**.0217**
	Bisphosphonate * Stage G2 CKD (eGFR 60-89 mL/min/1.73 m^2^)	0.003514	0.003123	.2605
	Bisphosphonate * Stage G3A CKD (eGFR 45-59 mL/min/1.73 m^2^)	0.005693	0.004021	.1569
	Bisphosphonate * Stage G3B CKD (eGFR 30-44 mL/min/1.73 m^2^)	−0.01850	0.01150	.1077
	Bisphosphonate * Stage G4 CKD (eGFR 15-29 mL/min/1.73 m^2^)	**0.2043**	**0.04468**	**<.0001**
eGFR model	Placebo (reference)			
	Bisphosphonate	**0.03093**	**0.006048**	**<.0001**
	eGFR	−0.00009	0.000073	.2066
	Bisphosphonate * eGFR	−0.00005	0.000078	.5366

*Note*. CKD = chronic kidney disease; eGFR = estimated glomerular filtration rate; SE = standard error.

*N* = 2617.

Bold values signify *p* < 0.05.

### Hypocalcemia

The mean follow-up time was 2.2 ± 1.0 years, and there were a total of 388 events in 7428 individuals with serum calcium < 2.00 mmol/L. In Cox PH models, bisphosphonate therapy increased the risk of hypocalcemia (HR 1.324, 95% CI 1.056, 1.660), but there was no evidence of any effect modification by CKD stage (all tests of interaction *p* > .05, Table 5, Supplemental Table 4).

**Table 5.** Cox PH Models for Hypocalcemia (Ca < 2.00 mmol/L).

	Treatment	CKD	Hazard ratio	95% CI	
CKD models	Placebo	Stage G1 CKD (eGFR >90 mL/min/1.73 m^2^) (reference)			
		Stage G2 CKD (eGFR 60-89 mL/min/1.73 m^2^)	0.733	0.396	1.360
		Stage G3A CKD (eGFR 45-59 mL/min/1.73 m^2^)	**0.481**	**0.238**	**0.969**
		Stage G3B CKD (eGFR 30-44 mL/min/1.73 m^2^)	1.219	0.456	3.255
		Stage G4 CKD (eGFR 15-29 mL/min/1.73 m^2^)	n/a	n/a	n/a
	Bisphosphonate	Stage G1 CKD (eGFR >90 mL/min/1.73 m^2^) (reference)			
		Stage G2 CKD (eGFR 60-89 mL/min/1.73 m^2^)	0.743	0.500	1.103
		Stage G3A CKD (eGFR 45-59 mL/min/1.73 m^2^)	0.766	0.499	1.176
		Stage G3B CKD (eGFR 30-44 mL/min/1.73 m^2^)	1.267	0.657	2.445
		Stage G4 CKD (eGFR 15-29 mL/min/1.73 m^2^)	n/a	n/a	n/a
	Bisphosphonate versus Placebo	Stage G1 CKD (eGFR >90 mL/min/1.73 m^2^)	1.172	0.622	2.208
		Stage G2 CKD (eGFR 60-89 mL/min/1.73 m^2^)	1.187	0.883	1.595
		Stage G3A CKD (eGFR 45-59 mL/min/1.73 m^2^)	**1.869**	**1.177**	**2.968**
		Stage G3B CKD (eGFR 30-44 mL/min/1.73 m^2^)	1.219	0.463	3.208
		Stage G4 CKD (eGFR 15-29 mL/min/1.73 m^2^)	n/a	n/a	n/a
		All stages of CKD	**1.335**	**1.066**	**1.672**
eGFR models	Placebo	eGFR	1.005	0.991	1.018
	Bisphosphonate	eGFR	1.000	0.991	1.009
	Bisphosphonate vs Placebo	Placebo (reference)			
		Bisphosphonate^a^	**1.324**	**1.056**	**1.660**

*Note*. CKD = chronic kidney disease; eGFR = estimated glomerular filtration rate; CI = confidence interval.

a At mean eGFR 69 mL/min/1.73 m^2^.

Bold values signify *p* < 0.05.

*N* = 7428, 388 events.

### Decline in Kidney Function

**Figure 2.**
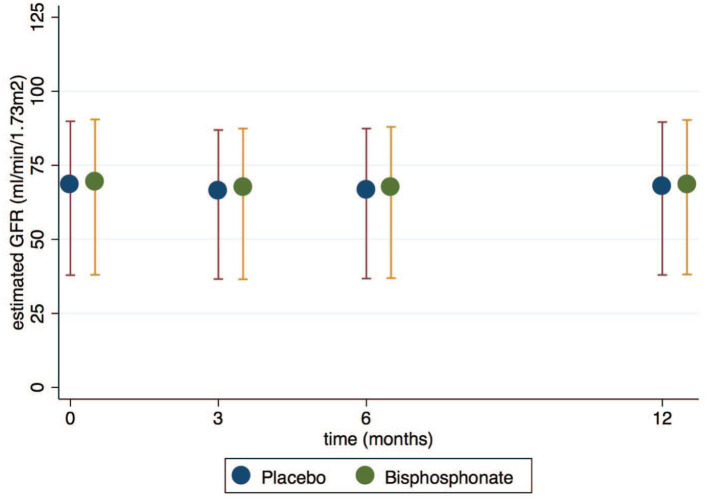
The effect of bisphosphonate versus placebo on eGFR over 12 months.

The mean (SD) baseline eGFR in the bisphosphonate group was 69.4 (16.0) and 68.4 (15.6) in the placebo group. There were 7428, 6884, 6766, and 6436 with serum creatinine measured at baseline, 3, 6, and 12 months, respectively. At 3, 6, and 12 months, there was no evidence of a change in eGFR between bisphosphonate and placebo groups (Figure 2 and Table 6). We did not compare rates of acute kidney injury defined by relative or absolute increases in serum creatinine given the lack of any change in eGFR longitudinally. No study reported albuminuria or proteinuria as outcomes.

**Table 6.** Multilevel Linear Regression Model for Serum Creatinine.

	β	SE	*P* value
Placebo (reference)			
Bisphosphonate	0.08365	0.2847	.7689
Placebo * months	**−0.1261**	**0.04524**	**.0053**
Bisphosphonate * months	**−0.1273**	**0.04360**	**.0035**

*Note*. SE = standard error.

*N* = 7428, 6884, 6766, and 6436 at baseline, 3 months, 6 months, and 12 months, respectively.

Bold values signify *p* < 0.05.

## Discussion

In this systematic review and individual patient meta-analysis of 7 randomized placebo-controlled trials of ibandronate that included 7428 participants with a mean (SD) eGFR was 69.1 (15.9) including 27.5% with stage G3A CKD and 3.0% of participants with stage 3B CKD, bisphosphonate therapy increased BMD at the hip and lumbar spine across the spectrum of CKD and non-significantly decreased the risk of vertebral and non-vertebral fractures except in stage G3B CKD where it increased the risk of fracture despite its positive effects on BMD. There was no effect modification of the risk of hypocalcemia with bisphosphonate therapy by CKD and there was no difference in eGFR between ibandronate and placebo over 1 year of follow-up across CKD stages.

The efficacy and safety of bisphosphonates in CKD have been explored in previous studies and have generally demonstrated efficacy with regard to BMD and fracture outcomes and overall safety but have typically not included individuals with advanced CKD and dialysis.^18,19^ Our finding of an increased fracture risk in stage G3B CKD has not been previously reported but needs to be interpreted in the totality of evidence of all agents in this class of anti-resorptive agents. An analysis of a database containing 9 phase 3 randomized, double-blind, placebo-controlled trials of risedronate for osteoporosis therapy in women comprising 9883 patients showed that CKD was common despite the exclusion of patients with Cr>1.1 times upper limit of normal: 48% had mild CKD (CrCl≥50 to <80 mL/min), 45% had moderate CKD (CrCl ≥ 30 to <50 mL/min), and 7% had severe CKD (CrCl < 30 mL/min). However, adverse events and kidney function were similar in all groups regardless of kidney function with no differences in efficacy.^19^ A post-hoc analysis of the Fracture Intervention Trial showed that 9.9% of the study’s 6458 participants had a CrCl ≤45 mL/min and 37.3% had a CrCl 45 to 59 mL/min. Patients treated with alendronate with CKD had an increased total hip BMD but no differences at other sites, clinical fractures, vertebral fractures, or adverse events compared with those without CKD.^18^ Other population-based studies also support the safety of bisphosphonates in CKD. In a population based-cohort of over 120 000 adults from 1996 to 2009 in Ontario, Canada, in which oral bisphosphonates were prescribed for those 66 years or older with new fragility fractures, there was no association of bisphosphate use with 90-day AKI hospitalizations or nephrology consults even in subgroups with CKD (23.6%, 13.5%, and 4.1% stages 3A, 3B, and 4, respectively).^20^ In a retrospective cohort study of adult women including 9604 with stage 3 or 4 CKD with a mean eGFR of 51.8 mL/min/1.73 m^2^ (SD 10.7) and 89.2% with a history of osteoporosis, osteopenia, fracture or falls, bisphosphate therapy was associated with an adjusted HR of 0.78 (95% CI 0.67, 0.91) for death with no difference in a composite of cardiovascular events.^21^

Our results may differ from that of these 2 previous large studies due to possible differences in patient populations beyond simply CKD and GFR or pharmacologic differences between ibandronate and both alendronate or risedronate.^22^ Its results are also fragile^23^ as only 3.0% of participants had stage G3B CKD with limited fracture events compared with the 2 other studies where 9.9% had a CrCl < 45 mL/min and 45% had a CrCl of 30 to <50 mL/min. The finding that individuals with stage G3B CKD may be at an increased risk of fracture with the use of bisphosphonate therapy compared with placebo despite improvements in BMD may be due to underlying adynamic bone disease and the development of atypical fractures given the mean (SD) baseline PTH was 4.3 pmol/L (1.9), but this was only reported in 499/7428 (6.7%) of participants.^24^ Ibandronate has specifically been evaluated in the setting of CKD and dialysis and is known to be both renally cleared and dialyzable^25,26^ but studies are generally small and do not have long-term follow-up. From kidney and safety perspectives, ibandronate 3 mg injection/infusion every 3 months has been shown to be no different from alendronate 70 mg by mouth every week at 1 year in the DIVINE study which included women with postmenopausal osteoporosis at increased risk of kidney disease with at hypertension, diabetes or GFR < 60 mL/min/1.73 m^2^.^27^ In a retrospective observational study that included 7 adults on hemodialysis with a femoral neck T-score less than –2.5 and bone biopsy showing osteitis fibrosa and an iPTH < 300 pg/mL, ibandronate 1 mg IV every month did not improve BMD at a mean follow-up of 13 months. However, teriparatide which was used as an anabolic treatment for adynamic bone disease, did improve BMD.^28^ However, in an open-label study of 16 adults with kidney failure treated with hemodialysis with low BMD (mean lumbar T-score –3.01 (SD 1.11) and elevated PTH > 2x upper limit of normal, ibandronate 2 mg IV every 4 weeks decreased bone turnover markers and increased lumbar BMD measured by quantitative computed tomography at 48 weeks.^29^

This study has important clinical and research implications. From a clinical perspective, there is an urgent unmet need for treatments that reduce osteoporosis/osteopenia fracture risk in patients with CKD and kidney failure. The current approach of relying on bone turnover biomarkers to guide therapy with or without bone biopsy^30^ is not ideal as biomarkers only have moderate accuracy in predicting bone turnover and underlying bone histopathology.^31-33^ In addition, bone biopsies are not readily available in many centers and even if they are assessable, they are typically avoided because they are invasive, painful, and costly. This has resulted in the default position of not offering bisphosphonates (or in fact many anti-resorptive treatments) in patients with eGFR < 30 mL/min/1.73 m^2^ due to uncertainty regarding their efficacy and safety. From a research perspective, it is clear that the nephrology community needs to prioritize advancing the intersection of CKD and kidney failure and osteopenia/osteoporosis. This individual patient-level meta-analysis, although disappointing with only data from 7 of 39 possible studies, is an attempt at one of the many studies that need to be conducted in this area. Ultimately, we require a multicenter prospective observational study of adults with osteoporosis/osteopenia from across the spectrum of advanced CKD and kidney failure treated with bisphosphonates and other anti-resorptive treatments with serial BMD measurements (DEXA, quantitative CT, and MRI), bone turnover biomarkers, and bone biopsy histopathology that could address many of the remaining questions regarding non-invasive monitoring for adynamic bone disease. Large, randomized placebo-controlled trials of bisphosphonates or other anti-resorptive agents are needed in CKD and dialysis to determine their efficacy and safety which will require an enormous amount of effort and collaboration globally.^34-36^

The strengths of this study include its search strategy, size, use of individual patient-level data, and stratification of effect sizes and harms by CKD subgroups and eGFR subgroups. It is one of the largest studies to date dedicated to the efficacy and safety bisphosphonate therapy in CKD. However, it has its limitations. First, of the 39 studies identified in the systematic review, only 7 provided individual patient-level data (all through clinicalstudydatarequest.com and by Roche/GlaxoSmithKline) and ibandronate was the only bisphosphonate included in the meta-analysis. Whether our results are generalizable to bisphosphonates other than ibandronate is unclear but their efficacy and safety are presumably agnostic of the specific agent within this drug class. Second, included trials exclusively recruited women that were mostly white and postmenopausal so whether our results apply to other patient populations and forms of osteopenia/osteoporosis is unknown. Thirdly, participants with advanced CKD (ie, stage G4 and G5 CKD) that are more likely to have CKD-MBD and toxicity related to bisphosphonate accumulation were not represented but the effect modification for fracture seen in stage G3A CKD is notable. However, this finding is fragile and may be due to imbalances in prognostic factors and confounding due to its small sample size despite randomization. It is noted that randomized controlled trials (RCTs) of bisphosphonates in advanced CKD and dialysis do exist but are limited to small trials focused on cardiovascular surrogate outcomes.^35,37^ Fourth, we did not examine bone turnover markers or bone biopsy histopathology. Fifth, we were unable to analyze the risk of specific harms including osteonecrosis, atypical fractures, and arrhythmias, given their rarity and heterogeneity in classifications across studies with no long-term follow-up RCTs for safety. Finally, we did not focus on other anti-resorptive or anabolic therapies such as denosumab,^38^ raloxifene,^39^ teriparatide,^40^ and abaloparatide^41^ or romosozumab.^42^

## Conclusions

In summary, in a systematic review and individual patient meta-analysis, we found that CKD did not modify the benefit of ibandronate on the increase in hip and spine BMD at 1 year in adult women with osteoporosis or osteopenia or the risk of hypocalcemia and that ibandronate was safe from a kidney perspective. It did, however, increase the risk of fractures in patients with stage G3 CKD but this is not consistent with previous literature in RCTs of alendronate or risedronate. Patients with advanced CKD and kidney failure are not represented in randomized placebo-controlled bisphosphonate trials. Additional research is needed to determine the efficacy and safety of bisphosphonates in CKD and kidney failure as well as the role of other anti-resorptive agents and the utility of bone turnover markers to guide their appropriate use.

## Supplemental Material

sj-docx-1-cjk-10.1177_20543581241283523 – Supplemental material for The Efficacy and Safety of Bisphosphonate Therapy for Osteopenia/Osteoporosis in Patients With Chronic Kidney Disease: A Systematic Review and Individual Patient-Level Meta-Analysis of Placebo-Controlled Randomized Trials Supplemental material, sj-docx-1-cjk-10.1177_20543581241283523 for The Efficacy and Safety of Bisphosphonate Therapy for Osteopenia/Osteoporosis in Patients With Chronic Kidney Disease: A Systematic Review and Individual Patient-Level Meta-Analysis of Placebo-Controlled Randomized Trials by Reid Whitlock, Kerry MacDonald, Navdeep Tangri, Michael Walsh and David Collister in Canadian Journal of Kidney Health and Disease
