# Supplementary material for: The Efficacy and Safety of Bisphosphonate Therapy for Osteopenia/Osteoporosis in Patients With Chronic Kidney Disease: A Systematic Review and Individual Patient-Level Meta-Analysis of Placebo-Controlled Randomized Trials
Source: Can J Kidney Health Dis. 2024 Oct 8;11:20543581241283523. doi: 10.1177/20543581241283523 (PMC11459530; doi:10.1177/20543581241283523)

**Supplemental Material**

Supplemental Table 1: Characteristics of Included Studies

| Study |  | Population | Exclusion | Therapy | Supplementation | Follow-up | Assessment | | | Outcome |
| --- | --- | --- | --- | --- | --- | --- | --- | --- | --- | --- |
| 1^st^ author  Date of publication  Dates | Industry | Number  Age  Sex  BMD  other | Definition of renal dysfunction | Drug  Comparator | Calcium (mg)  Vitamin D (IU)  Concomitant therapy | Planned  Median F/U  Mean F/U  % completed | Fractures | BMD | Other | Primary |
| Watts(1)  (1990)  N/A | P&G | N =423  <75 years  women  radiographic evidence of vertebral osteopenia  at least 1 but not more than 4 vertebral compression fractures  N/A | SCr>210μmol/L | Phosphate 1g po bid or placebo for 3 days then etidronate 400mg po daily or placebo for 14 days then calcium 500mg po daily for 74 days for 8 91 day cycles | 500mg po daily for 74 days  none  N/A | 2 years  N/A  N/A  86% | Lateral T, L spine x-rays at baseline, 12, 24 months | LS, H, R, at baseline, 6, 12, 18, 24 months | Bone biopsy at 2 years in selected individuals | N/A |
| Harris(2)  (1993)  N/A | P&G | N = 423  <75 years  postmenopausal women  N/A  1-4 vertebral fractures  N/A | N/A | P 2g po daily vs placebo x 3 days, ETI 400mg po daily vs placebo x 14 days, Ca 500mg po daliy x 74 days, repeat cycle N/ | N/A  N/A  N/A | 3-5 years  N/A  N/A  86% at 2 years, 95% at 3 years, 99% at 4 years | Lateral T, L spine x-rays at baseline, q yearly | LS, PF (FN, TR, WT) at baseline, q6 months | N/A | N/A |
| Adami(3)  (1995)  N/A | Merck | N = 286  48-76 years  postmenopausal females  >2 SD below the mean for young premenopausal females  N/A  N/A | N/A | aledronate 10mg po daily vs 20mg po daily vs intranasal calcitonin 100IU daily vs placebo | 500  N/A | 2 years  N/A  N/A  N/A | N/A | LS, H at baseline, 6,12,18,24 months | BTM at baseline, 3, 6, 9, 12, 15, 18, 21, 24 months | % change in LS BMD at 24 months |
| Liberman(4)  (1995)  N/A | Merck | N = 994  45-80 years  postmenopausal women  lumbar spine T-score <-2.5 SD  with or without fractures | SCr>130μmol/L | ALE 5mg po daily vs 10mg po daily vs 20mg po daily vs placebo but at 24 months 20mg switched to 5mg | 500  none | 3 years  N/A  N/A  91% at 1 year | Lateral spine x-rays at baseline, 12, 24, 36 months | LS, FN, TR, F, TB at baseline, 3, 6, 12, 18, 24, 30, 36 months | Height at baseline, 3, 6, 9, 12, 18, 24, 27, 30, 36 months, symptomatic fractures | % change in LS, FN, TR, F, TB BMD at 24 months |
| Tucci(5)  (1996)  N/A | Merck | N = 478  45-82 years  postmenopausal women  lumbar spine T-score -2.5SD or less  N/A  N/A | SCr>1.5mg/dL | ALE 5mg po daily vs 10mg po daily vs 20mg po daily vs placebo but at 24 months 20mg switched to 5mg | 500  none  N/A | 3 years  N/A  N/A  N/A | Lateral T, L spine –x-rays at baseline, 12, 24, 36 months | LS, FN, TR, TH, F, TB at baseline, 3, 6, 9, 12, 15, 18, 24, 27, 30, 33, 36 months | Height at baseline, q3-6 monthly  BTM at baseline, q3-6 monthly, bone biopsy at 3 years in selected individuals | % change in LS BMD at 12, 24, 36 months |
| Black(6)  (1996)  N/A  FIT | Merck | N = 2027  55-81 years  postmenopausal women  femoral neck <2.1 below peak bone mass  vertebral fracture | SCr>144μmol/L | ALE 5mg po daily increased to 10mg po daily at 24 months vs placebo | 500  250  N/A | 3 years  N/A  2.9 years  96.2% | Lateral x-rays at baseline, 24, 36 months | H, PA and lateral LS, 20% F at baseline, 12, 24, 36 months  TB at baseline, 36 months | N/A | vertebral fractures at 36 months |
| Devogelaer(7)  (1996)  N/A | Merck | N = 516  45-80 years  postmenopausal women  at least 2.5 SD below the mean for young premenopausal women  no more than 1 lumbar fracture, no femoral neck fracture  N/A | N/A | ALE 5mg po daily vs 10mg po daily vs 20mg po daily (5mg po daily at 2 years) vs placebo | 500  none  N/A | 3 years  N/A  N/A  N/A | N/A | LS, FN, TR, F, TB at baseline, 3, 6, 12, 18, 24, 36 months | Stature and BTM at 1, 3, 6, 12, 18, 24, 30, 36 months | LS BMD at 36 months |
| Bone(8)  (1997)  N/A | Merck | N = 359  60-85 years  postmenopausal women  lumbar spine 2.0 SD below mean peak levels  excluded if more than 1 lumbar fracture  N/A | N/A | ALE 1.0mg po daly vs 2.5mg po daily vs 5.0mg po daily vs placebo | 500  none  N/A | 2 years  N/A  N/A  N/A | Lateral T, L spine x-rays at baseline, 12, 24 months  Non-vertebral fractures by clinical, x-rays | LS, FN, GT, WT at baseline, 3, 6, 9, 12, 24 months | BTM q3-6 months, bone biopsy at 1 or 2 years | BMD |
| Harris(9)  (1999)  1993-1998  VERT | P&G, Hoechst Marion Roussel | N = 2458  <85 years  postmenopausal women  2 or more vertebral fractures or 1 vertebral fracture and low L-spine BMD with T-score -2 SD or less | N/A | RIS 2.5mg po daily but discontinued vs RIS 5mg po daily vs placebo | 1000  <500  NA | 3 years  N/A  N/A  57.7% | Lateral T, L spine x-rays at baseline, 12, 24, 36 months  Non-vertebral fractures | LS, FN at baseline, 6, 12, 18, 24, 30, 36 months | N/A | New, worsening vertebral fractures |
| Pols(10)  1999  N/A  FOSIT | Merck | N=1908  <85 years  postmenopausal women  lumbar spine at least 2 SD below the mean for mature, premenopausal females  N/A  N/A | SCr>150micromol/L | ALE 10mg po daily versus placebo | 500  <1000 but not provided | 1 year  N/A  N/A  89.0% | Clinical fractures through adverse event reporting | LS, FN, TR, TH at baseline, 3, 6, 12 months | BTM at each clinic visit | LS BMD |
| Lau(11)  (2000)  N/A | N/A | N = 100  60-79 years  postmenopausal women  lumbar spine T-score <2.5  no history of hip fracture  N/A | Abnormal renal function | ALE 10mg po daily vs placebo | 500  none  N/A | 1 year  N/A  N/A  78% | N/A | LS, FN, TR at baseline, 6, 12 months | N/A | N/A |
| Orwoll(12)  (2000)  N/A | Merck | N = 242  N/A but 31-87 years  Male  Femoral neck T-score<-2D and lumbar spine T-score<-1SD or BMD at femoral neck <-SD and at least 1 vertebral deformity or history of osteoporotic fracture | SCr>144μmol/L | ALE 10mg po daily versus placebo | 500  400-450  N/A | 2 years  N/A  N/A  85% | PA and lateral x-rays of T, L spine at baseline, 24 months  Non-vertebral fractures | LS, TH, TB at baseline, 6, 12, 18, 24 months | Height at baseline, 3, 6, 12, 18, 24 months | LS BMD |
| Fogelman(13)  2000  N/A | P&G, Aventis | N = 543  <80 years  postmenopausal women  lumbar spine T-score <-2  N/A  N/A | N/A | RIS 2.5mg po daily vs RIS 5mg po daily vs placebo | 1000  none  N/A | 2 years  N/A  N/A  65.4% | Lateral and AP x-rays of T, L spine at baseline, 24 months | LS, FN, TR at baseline, 6, 12, 18, 24 months | BTM but not timeslines | LS BMD at 24 months |
| Reginster(14)  (2000)  N/A  VERT | P&G, Hoechst Marion Roussel | N = 1226  <85 years  postmenopausal women  N/A  At least 2 vertebral fractures  N/A | N/A | RIS 2.5mg po daily (D/C at 2 years) vs RIS 5.0mg po daily vs placebo | 1000  up to 500  N/A | 3 years  N/A  N/A  44.4% | Lateral TL spine x-rays at baseline and 12, 24, 36 months | BMD at baseline, 6, 12, 18, 24, 30, 36 months | BTM at baseline, 1, 3, 6, 12, 24, 36 months | Incidence of vertebral fractures over 3 years |
| Reginster(15)  (2001)  1992-1994 | Sanofi | N = 2293  Non-Fx N=488  Fx N = 1805  50-80 years  postmenopausal women  non-Fx study: lumbar spine T-score <-2  Fx study: at least 2 mild or 1 moderate but no more than 5 vertebral fractures and lumbar spine T-score<-1  N/A | N/A | TIL 50mg po daily x 7 days q montly vs TIL 200mg po daily x 7 days q monthly vs placebo | 500  none  N/A | Non-Fx: 2 years  Fx: 3 years  N/A  N/A  Non-Fx: 86.9%  Fx: 77.0% | Lateral T, L spine x-rays at baseline, 12, 24, 36 months | N/A | N/A | Non-Fx: % change from baseline BMD of AP LS at 2 years  Fx: proportion of patients who had vertebral fractures over 3 years |
| Riis(16)  (2001)  N/A | F. Hoffmann-La Roche | N =240  55-76 years  postmenopausal women  LS or FN T-score below -2.5SD  N/A  N/A | SCr>210micromol/L (2.4mg/dL) | IBA 2.5 mg IV daily vs IBA 20mg IV every other day for 24 days every 3 months vs placebo | 500  400  N/A | 2 years  N/A  N/A  78.3% | N/A | BMD at baseline, 3, 6, 9, 12, 15, 18, 21, 24 months | N/A | % change in BMD at LS at 12, 24 months |
| McClung(17)  (2001)  1993-1998 | P&G, Aventis | Group 1  N = 5445  70-79 years  postmenopausal females  femoral neck T-score less than -4 SD or -3 SD plus at least 1 risk factor for hip fracture  N/A  Group 2  N = 3886  >80 years  postmenopausal women  femoral neck T-score less than -4.5 SD or -3 SD plus hip-axis length >11cm or at least one non-skeletal risk factor  N/A | “important abnormalities in the results of routine laboratory tests” | RIS 2.5mg or 5mg po daily vs placebo | 100-400-0mg  <500  10.5% | 3 years  N/A  2.3 years  64% | N/A | BMD at baseline, 6, 12, 18, 24, 30, 36 months | N/A | Radigraphically confirmed hip fractures |
| Greenspan(18)  (2002)  N/A | Merck | N = 327  >65 years  females  T-score lower than -2.0 SD of lumbar spine or hip  N/A  ambulatory, long-term care | N/A | ALE 10mg po daily vs placebo | 500  400  N/A | 2 years  N/A  N/A  N/A | N/A | N/A | N/A | N/A |
| Reid(19)  (2002)  N/A | Novartis | N =351  45-80 years  postmenopausal women  lumbar spine T-score lower than -2SD  no more than 1 vertebral fracture  N/A | renal disease | ZOL 0.25mg vs ZOL 0.5mg vs ZOL 1mg IV q3 months, ZOL 2mg q6months, ZOL 4mg q yearly | 1000  none  N/A | 1 year  N/A  N/A  90.0% | N/A | LS, non-dominant PF, F, TB at baseline, 6, 9, 12 months | BTM | N/A |
| Valimaki(20)  (2002)  N/A | Leiras Oy | N=610  >45 years  postmenopausal women  lumbar spine T-score <1 SD  N/A  N/A | SCr>120micromol/L | CLO 65mg po daily vs CLO 400mg po daily vs 800mg po daily vs 400mg 15/90 days vs placebo | N/A  <400 but no assigned  N/A | 3 years  N/A  N/A  83.4% | T, L spine x-rays at baseline, 36 months | LS, PF at 0, 24, 36 months | BTM, bone biopsy at 3 years in selected volunteers | % change in BMD of LS and FN, TR, WT |
| Hosking(21)  (2003) | Merck | N = 549  >60 and <90 years  post-menopausal women  BMD lumbar spine or total hip <-2.5 or both lumbar spine and total hip T-score <2.0  N/A  “patients with osteoposis, so severe that (in the judgment of the investigator) participation in a placebo-control trial was unethical, were excluded” | N/A | ALE 70mg po q weekly vs RIS 5mg po daily vs placebo | 1000 (but total intake)  400 (if 25-OH vitamin D<15ng/ml) | 1 year  N/A  N/A  80.0% at 12 weeks | N/A | LS, FN, TR, TH at baseline, 6, 12 months | BTM at baseline, 1,3,6,12 months | % change in NTX at 3 months |
| Stakkestad(22) (2003)  1998-2000 | Hoffmann-La Roche | N = 627  N/A  Postmenopausal women  4 strata by lumbar spine BMD and menopause: T score >-1 1-3 years, <-1 and >2.5 and 1-3 years, >-1 and >3 years, <-1 and >2.5 and >3 years but not <2.5 SD of osteoporotic fractures  N/A | SCr>201micromol/L | IBA 0.5mg IV q3 montly vs 1.0mg IV q3 monthly vs 2.0mg IV q3 monthly vs placebo | 500  none  N/A | 1 year  N/A  N/A  90.3% | N/A | LS, PF (TH, FN, TR) at baseline, 6, 12 months | BTM at baseline and subsequent visits at selected centers | Relative change in LS BMD at 12 months |
| Delmas(23)  (2004)  1996-2000  BONE  2004 | Hoffmann-La Roche | N = 2946  55-80 years  postmenopausal women  lumbar T-score -2.0 to -5.0  1-4 vertebral fractures  N/A | NR | IBA 2.5mg po daily vs IBA 20mg po EOD x 12 doses q3 months vs placebo | 500  400  N/A | 3 years  N/A  N/A  ??? | T, L spine x-rays at baseline, 12, 24, 36 months | baseline, 6, 12, 18, 24, 36 months | BTM at baseline, 3, 6, 12, 18, 24, 36 months | morphometric vertebral fractures at 36 months |
| Adami(24)  (2004)  N/A | Hoffmann-La Roche | N = 520  55-75 years  postmenopausal women  lumbar spine T score<-2.5  N/A  N/A | SCr>210μmol/L | 1mg IBA IV q 3 months vs 2 mg IBA IV q 3 months vs placebo | 500  400 | 1 year  N/A  N/A  89.0% | N/A | LS, PFT, TB BMD at baseline, 6, 12 months | BTM at baseline, 3, 6, 9, 12 months | Relative change in LS BMD at 12 months |
| Miller(25)  (2004)  N/A  STRONG | Merck | N = 167  25-90 years  men  T-score at least -2 SD at femoral neck and at least -1SD at lumbar spine or T-score at least -2SD at lumbar spine and at least -1SD at lumbar spine or osteoporotic fracture and femoral neck T-score at least -1SD  idiopathic and hypogonadism  N/A | N/A | ALE 70mg po q weekly vs placebo | 500  200  N/A | 1 year  N/A  N/A  85.6% | Lateral spine x-rays at baseline, 12 months or early discontinuation | LS, TH, TB at baseline, 6, 12 months | BTM at baseline, 3,6,12 months | N/A |
| Recker(26)  (2004)  N/A | Hoffmann-La Roche | N = 2862  55-76 years  postmenopausal women  T score -2.0 to -5.0 in atleast 1 vertebra of LS  1-4 prevalent vertebral fractures  N/A  N/A | SCr>210micrmol/L | IBA 0.5mg IV q 3months vs IBA 1mg IV q 3 months vs placebo | 500  400  N/A | 3 years  N/A  N/A  82.4% | Lumbar spine x-rays at baseline, 12, 24, 36 months  Clinical fractures identified symptomatically, confirmed radiographically | BMD at baseline, 6, 12, 18, 24, 36 months | BTM at baseline, 3, 6, 12, 18, 24, 36 months  Height at baseline, 3, 6, 9, 12, 15, 18, 21, 24, 27, 30, 33, 36 months | New incident vertebral fractures at 3 years |
| Bonnick(27)  (2007)  1995-1998 | Merck | N = 701  >45 years and >18 years if surgical menopause  postmenopausal women  L1-L4 >2 SD below peak BMD  N/A  community dwelling | CrCl<35 or SCr 1.6mg/dL | ALE 10mg po daily/calcium placebo vs ALE 10mg po daily/calcium 1000mg po daily vs ALE placebo/calcium 100mg po daily | intervention  400  N/A | 2 years  N/A  N/A  89.0% | N/A | LS, FN, TR at baseline, 6, 12, 24 months | BTM at 3, 6, 12, 24 months  Height at baseline, 12 months | % change in BMD of PA LS at 24 months |
| Black(28)  (2007)  2002-2006  HORIZON-PFT | Novartis | N = 7765  65-89 years  postmenopausal women  T score -2.5 SD or less or T score of -1.5 SD or less with radiologic evidence of 2 mild or 1 moderate vertebral fractures | CrCl<30 or urinalysis >2+ for protein | q yearly ZOL 5mg IV vs placebo | 1000-1500  400-1200  yes | 3 years  N/A  N/A  84% | Lateral spine x-rays at baseline, 12, 24, 36 months or early termination for stratum 1, baseline, 36 months for stratum 2, clinical fractures with imaging, surgery | TH, FN, LS at 0, 6, 12, 24, 36 months | N/A | new vertebral fractures, hip fractures |
| Lyles(29)  (2007)  N/A  HORIZON-RFT | Novartis | N = 2127  >50 years  men or women  N/A  hip fracture sustained with minimal trauma | CrCl<30 | q yearly ZOL vs placebo | 1000-1500  800-1200  yes | 5 years  1.9 years  N/A  71.3% | hip x-ray at baseline, 1, 2, 3, 4, 5 years | TH, FN at baseline, 1, 2, 3, 4, 5 years | N/A | new clinical fracture excluding facial, digital, pathologic fracture |
| Valimaki(30)  (2007)  N/A | P&G, Sanofi-Aventis | N=171  N/A  Postmenopausal women  Lumbar spine T-score between  -2.5 and -1 SD  without >2 lumbar fractures  ambulatory, >1 risk factor for osteoporosis | N/A | RIS 5mg po daily vs placebo | 1000  400  N/A | 2 years  646.6 (207.9) days for RIS, 622.9 (230.8) days for placebo  N/A | clinical vertebral and non-vertebral fractures | LS, F at baseline, 12, 24 monhts | BTM at baseline, 12, 24 months | % changes in LS BMD at 24 months |
| Boonen(31)  (2009)  N/A | P&G, Sanofi-Aventis | N = 284  >30 years  men  lumbar spine T-score <2.5 and femoral neck T-score <1 or lumbar spine T-score <1 and femoral neck T-score <2  <1 osteoporotic fracture <6 months ago (amendment)  ambulatory, primary hypogonadism if declined testosterone therpay | N/A | RIS 35mg po q weekly vs placebo | 1000  400-500  N/A | 2 years  N/A  N/A  91.2% | Lateral T, L spine x-rays at baseline, 12, 24 months, clinical fractures | LS, PF (TH, FN, TR) at baseline, 6, 12, 24 months | BTM at baseline, 3, 6, 12, 24 months, height at baseline, 12, 24 months | % change in LS BMD at 24 months |
| McClung(32)  (2009)  N/A | Roche | N = 160  45-60 years  postmenopausal women  lumbar spine T-score between -1.0 and -2.5 and proximal femur T score >-2.5  no fractures  ambulatory | Calculated GFR<30ml/min | IBA 150mg po q monthly vs placebo | 500  400  N/A | 1 year  N/A  N/A  86.3% | Fractures were confirmed by radiography and reported as adverse events | BMD but no timelines | BTM but no timelines | % change in LS BMD at 12 months |
| McClung(33)  (2009)  N/A | Novartis | N = 581  >45 years  postmenopausal women  T-score less than -1.0 and more than -2.5 a lumbar spine and a T-score greater than -2.5 at the femoral neck  <1 grade 1 vertebral fracture and no grade 2 or 3 vertebral fractures  N/A | CrCl<30ml/min | ZOL 5mg IV at baseline, 12 months vs ZOL 5mg IV at baseline, placebo at 12 months vs placebo | 500-1200  400-800  N/A | 2 years  N/A  N/A  90.0% | Lateral T, L x-rays at baseline, clinical fractures | BMD at baseline, 6, 12, 24 months | BTM at baseline, 1, 3, 6, 9, 12, 15, 18, 24 months | % change in LS BMD at 24 months |
| Matsumoto(34)  (2009)  N/A | Astellas, Ono | N = 704  55-80 years  postmenopausal women  lumbar BMD<1.7 SD  1-5 fragility fractures T4-L4  N/A | SCr>1.5mg/dL = 133 micromol/L | Minodronate 1mg po daily vs placebo | 600  200  N/A | 2 years  N/A  N/A  69.9% | Lateral T, L spine x-rays at baseline, 6, 12, 18, 24 months, clinical fractures | N/A | BTM at baseline, 6, 12, 18, 24 months | new morphometic vertebral fractures at 24 months |
| Yan(35)  (2009)  N/A | CSPC Group, Hebei | N=560  <85 years  postmenopausal women  lumbar spine T-score<2  no vertebral fractures  ambulatory  N/A | Renal dysfunction | ALE 70mg po qweekly vs placebo | 500  200  N/A | 1 year  N/A  N/A  80.2% | N/A | LS, FN, TR, TH at baseline, 6, 12 months | BTM at baseline, 6, 12 months | % change in LS BMD at 12 months |
| Boonen(36)  (2012)  2006-2010  N/A | Novartis | N=1199  50-85 years  men  T score of -1.5 of less at the total hip or femoral neck and 1-3 prevalent vertebral fractures or T score of -2.5 or less at total hip, femoral neck or lumbapr spine  N/A  N/A | CrCl<30 | Q yearly 5mg ZOL vs placebo | 1000-1500  800-1200 | 2 years  N/A  N/A  89.2% | Lateral T, L spine x-rays at baseline, 12, 24 months  Clinical fractures by imaging, surgery | LS, TH, FN at baseline, 6, 12, 24 months in subgroup | BTM at baseline, 3, 6, 12, 15, 18, 24 months in subgroup, height at baseline, 12, 24 months | new morphometric vertebral fractures over 24 months |
| Chapurlat(37)  (2013)  2007-2010 | La Roche | N = 148  55-75 years  postmenopausal women  T score -1.0 to -2.5 SD at spine or hip  No grade 2,3 vertebral fractures, hip fractures  ambulatory  N/A | CrCl<30 | Q monthly IBA 150mg vs placebo | 500  400 | 2 years  N/A  N/A  99.3% | N/A | aBMD at LS, radius, TH | vBMD and microarchitecture at non-dominant radius, right tibia by HR-pQCT at baseline, 6, 12, 24 months, BTM | BV/TV at 12 months by HR-pQCT at non-dominant radius |
| Yang(38)  (2015)  N/A |  | N = 100  N/A  Postmenopausal women  T-score <2.5 at lumbar spine or total hip  N/A  N/A | N/A | ZOL 5mg IV x 1 vs placebo | 1000  400  N/A | 1 year  N/A  N/A  90% | N/A | LS, TH at baseline, 12 months | BTM at baseline, 12 months and proton MRS of vertebral body | N/A |
| Lewiecki(39) (2009)  N/A | GSK | N = 93  55-80 years  12 months without menses or 6 weeks after oophorectomy  BMD T-scores 2.0 or less (lumbar spine, total hip, or femoral neck) and -5.0 or greater (all sites)  Exclusion = vertebral fractures, hip implants, major GI lesions, diseases or medications affecting bone metabolism, or history of non-basal cell cancer | N/A | Ibandronate 150mg po once monthly vs placebo | 1000  400  N/A | 1 year  N/A  N/A  N/A | N/A | LS, TH DXA and QCT and at baseline, 12 months, | BTM at baseline, 3,6,9,12 months, FEA | TH QCT BMD |

References:

1. Watts NB, Harris ST, Genant HK, Wasnich RD, Miller PD, Jackson RD, Licata AA, Ross P, Woodson GC,3rd, Yanover MJ. Intermittent cyclical etidronate treatment of postmenopausal osteoporosis. N Engl J Med. 1990 Jul 12;323(2):73-9.

2. Harris ST, Watts NB, Jackson RD, Genant HK, Wasnich RD, Ross P, Miller PD, Licata AA, Chesnut CH,3rd. Four-year study of intermittent cyclic etidronate treatment of postmenopausal osteoporosis: Three years of blinded therapy followed by one year of open therapy. Am J Med. 1993 Dec;95(6):557-67.

3. Adami S, Passeri M, Ortolani S, Broggini M, Carratelli L, Caruso I, Gandolini G, Gnessi L, Laurenzi M, Lombardi A. Effects of oral alendronate and intranasal salmon calcitonin on bone mass and biochemical markers of bone turnover in postmenopausal women with osteoporosis. Bone. 1995 Oct;17(4):383-90.

4. Liberman UA, Weiss SR, Broll J, Minne HW, Quan H, Bell NH, Rodriguez-Portales J, Downs RW,Jr, Dequeker J, Favus M. Effect of oral alendronate on bone mineral density and the incidence of fractures in postmenopausal osteoporosis. the alendronate phase III osteoporosis treatment study group. N Engl J Med. 1995 Nov 30;333(22):1437-43.

5. Tucci JR, Tonino RP, Emkey RD, Peverly CA, Kher U, Santora AC,2nd. Effect of three years of oral alendronate treatment in postmenopausal women with osteoporosis. Am J Med. 1996 Nov;101(5):488-501.

6. Black DM, Cummings SR, Karpf DB, Cauley JA, Thompson DE, Nevitt MC, Bauer DC, Genant HK, Haskell WL, Marcus R, Ott SM, Torner JC, Quandt SA, Reiss TF, Ensrud KE. Randomised trial of effect of alendronate on risk of fracture in women with existing vertebral fractures. fracture intervention trial research group. Lancet. 1996 Dec 7;348(9041):1535-41.

7. Devogelaer JP, Broll H, Correa-Rotter R, Cumming DC, De Deuxchaisnes CN, Geusens P, Hosking D, Jaeger P, Kaufman JM, Leite M, Leon J, Liberman U, Menkes CJ, Meunier PJ, Reid I, Rodriguez J, Romanowicz A, Seeman E, Vermeulen A, Hirsch LJ, Lombardi A, Plezia K, Santora AC, Yates AJ, Yuan W. Oral alendronate induces progressive increases in bone mass of the spine, hip, and total body over 3 years in postmenopausal women with osteoporosis. Bone. 1996 Feb;18(2):141-50.

8. Bone HG, Downs RW,Jr, Tucci JR, Harris ST, Weinstein RS, Licata AA, McClung MR, Kimmel DB, Gertz BJ, Hale E, Polvino WJ. Dose-response relationships for alendronate treatment in osteoporotic elderly women. alendronate elderly osteoporosis study centers. J Clin Endocrinol Metab. 1997 Jan;82(1):265-74.

9. Harris ST, Watts NB, Genant HK, McKeever CD, Hangartner T, Keller M, Chesnut CH,3rd, Brown J, Eriksen EF, Hoseyni MS, Axelrod DW, Miller PD. Effects of risedronate treatment on vertebral and nonvertebral fractures in women with postmenopausal osteoporosis: A randomized controlled trial. vertebral efficacy with risedronate therapy (VERT) study group. JAMA. 1999 Oct 13;282(14):1344-52.

10. Pols HA, Felsenberg D, Hanley DA, Stepan J, Munoz-Torres M, Wilkin TJ, Qin-sheng G, Galich AM, Vandormael K, Yates AJ, Stych B. Multinational, placebo-controlled, randomized trial of the effects of alendronate on bone density and fracture risk in postmenopausal women with low bone mass: Results of the FOSIT study. fosamax international trial study group. Osteoporos Int. 1999;9(5):461-8.

11. Lau EM, Woo J, Chan YH, Griffith J. Alendronate prevents bone loss in chinese women with osteoporosis. Bone. 2000 Nov;27(5):677-80.

12. Orwoll E, Ettinger M, Weiss S, Miller P, Kendler D, Graham J, Adami S, Weber K, Lorenc R, Pietschmann P, Vandormael K, Lombardi A. Alendronate for the treatment of osteoporosis in men. N Engl J Med. 2000 Aug 31;343(9):604-10.

13. Fogelman I, Ribot C, Smith R, Ethgen D, Sod E, Reginster JY. Risedronate reverses bone loss in postmenopausal women with low bone mass: Results from a multinational, double-blind, placebo-controlled trial. BMD-MN study group. J Clin Endocrinol Metab. 2000 May;85(5):1895-900.

14. Reginster J, Minne HW, Sorensen OH, Hooper M, Roux C, Brandi ML, Lund B, Ethgen D, Pack S, Roumagnac I, Eastell R. Randomized trial of the effects of risedronate on vertebral fractures in women with established postmenopausal osteoporosis. vertebral efficacy with risedronate therapy (VERT) study group. Osteoporos Int. 2000;11(1):83-91.

15. Reginster JY, Christiansen C, Roux C, Fechtenbaum J, Rouillon A, Tou KP. Intermittent cyclic tiludronate in the treatment of osteoporosis. Osteoporos Int. 2001;12(3):169-77.

16. Riis BJ, Ise J, von Stein T, Bagger Y, Christiansen C. Ibandronate: A comparison of oral daily dosing versus intermittent dosing in postmenopausal osteoporosis. J Bone Miner Res. 2001 Oct;16(10):1871-8.

17. McClung MR, Geusens P, Miller PD, Zippel H, Bensen WG, Roux C, Adami S, Fogelman I, Diamond T, Eastell R, Meunier PJ, Reginster JY, Hip Intervention Program Study Group. Effect of risedronate on the risk of hip fracture in elderly women. hip intervention program study group. N Engl J Med. 2001 Feb 1;344(5):333-40.

18. Greenspan SL, Schneider DL, McClung MR, Miller PD, Schnitzer TJ, Bonin R, Smith ME, DeLucca P, Gormley GJ, Melton ME. Alendronate improves bone mineral density in elderly women with osteoporosis residing in long-term care facilities. A randomized, double-blind, placebo-controlled trial. Ann Intern Med. 2002 May 21;136(10):742-6.

19. Reid IR, Brown JP, Burckhardt P, Horowitz Z, Richardson P, Trechsel U, Widmer A, Devogelaer JP, Kaufman JM, Jaeger P, Body JJ, Brandi ML, Broell J, Di Micco R, Genazzani AR, Felsenberg D, Happ J, Hooper MJ, Ittner J, Leb G, Mallmin H, Murray T, Ortolani S, Rubinacci A, Saaf M, Samsioe G, Verbruggen L, Meunier PJ. Intravenous zoledronic acid in postmenopausal women with low bone mineral density. N Engl J Med. 2002 Feb 28;346(9):653-61.

20. Valimaki MJ, Laitinen K, Patronen A, Puolijoki H, Seppanen J, Pylkkanen L, Aranko SM, Sairanen S, Blafield H, Rekiaro M, Vaisanen K, Kormano M, Makinen L, Salmi J, Ala-Kaila K, Perttila J, Vesterinen K, Koivunoro K, Probone Study Group. Prevention of bone loss by clodronate in early postmenopausal women with vertebral osteopenia: A dose-finding study. Osteoporos Int. 2002 Dec;13(12):937-47.

21. Hosking D, Adami S, Felsenberg D, Andia JC, Valimaki M, Benhamou L, Reginster JY, Yacik C, Rybak-Feglin A, Petruschke RA, Zaru L, Santora AC. Comparison of change in bone resorption and bone mineral density with once-weekly alendronate and daily risedronate: A randomised, placebo-controlled study. Curr Med Res Opin. 2003;19(5):383-94.

22. Stakkestad JA, Benevolenskaya LI, Stepan JJ, Skag A, Nordby A, Oefjord E, Burdeska A, Jonkanski I, Mahoney P, Ibandronate Intravenous Study Group. Intravenous ibandronate injections given every three months: A new treatment option to prevent bone loss in postmenopausal women. Ann Rheum Dis. 2003 Oct;62(10):969-75.

23. Delmas PD, Recker RR, Chesnut CH,3rd, Skag A, Stakkestad JA, Emkey R, Gilbride J, Schimmer RC, Christiansen C. Daily and intermittent oral ibandronate normalize bone turnover and provide significant reduction in vertebral fracture risk: Results from the BONE study. Osteoporos Int. 2004 Oct;15(10):792-8.

24. Adami S, Felsenberg D, Christiansen C, Robinson J, Lorenc RS, Mahoney P, Coutant K, Schimmer RC, Delmas PD. Efficacy and safety of ibandronate given by intravenous injection once every 3 months. Bone. 2004 May;34(5):881-9.

25. Miller PD, Schnitzer T, Emkey R, Orwoll E, Rosen C, Ettinger M, Vandormael K, Daifotis A. Weekly oral alendronic acid in male osteoporosis. Clin Drug Investig. 2004;24(6):333-41.

26. Recker R, Stakkestad JA, Chesnut CH,3rd, Christiansen C, Skag A, Hoiseth A, Ettinger M, Mahoney P, Schimmer RC, Delmas PD. Insufficiently dosed intravenous ibandronate injections are associated with suboptimal antifracture efficacy in postmenopausal osteoporosis. Bone. 2004 May;34(5):890-9.

27. Bonnick S, Broy S, Kaiser F, Teutsch C, Rosenberg E, DeLucca P, Melton M. Treatment with alendronate plus calcium, alendronate alone, or calcium alone for postmenopausal low bone mineral density. Curr Med Res Opin. 2007 Jun;23(6):1341-9.

28. Black DM, Delmas PD, Eastell R, Reid IR, Boonen S, Cauley JA, Cosman F, Lakatos P, Leung PC, Man Z, Mautalen C, Mesenbrink P, Hu H, Caminis J, Tong K, Rosario-Jansen T, Krasnow J, Hue TF, Sellmeyer D, Eriksen EF, Cummings SR, HORIZON Pivotal Fracture Trial. Once-yearly zoledronic acid for treatment of postmenopausal osteoporosis. N Engl J Med. 2007 May 3;356(18):1809-22.

29. Lyles KW, Colon-Emeric CS, Magaziner JS, Adachi JD, Pieper CF, Mautalen C, Hyldstrup L, Recknor C, Nordsletten L, Moore KA, Lavecchia C, Zhang J, Mesenbrink P, Hodgson PK, Abrams K, Orloff JJ, Horowitz Z, Eriksen EF, Boonen S, HORIZON Recurrent Fracture Trial. Zoledronic acid and clinical fractures and mortality after hip fracture. N Engl J Med. 2007 Nov 1;357(18):1799-809.

30. Valimaki MJ, Farrerons-Minguella J, Halse J, Kroger H, Maroni M, Mulder H, Munoz-Torres M, Saaf M, Snorre Ofjord E. Effects of risedronate 5 mg/d on bone mineral density and bone turnover markers in late-postmenopausal women with osteopenia: A multinational, 24-month, randomized, double-blind, placebo-controlled, parallel-group, phase III trial. Clin Ther. 2007 Sep;29(9):1937-49.

31. Boonen S, Orwoll ES, Wenderoth D, Stoner KJ, Eusebio R, Delmas PD. Once-weekly risedronate in men with osteoporosis: Results of a 2-year, placebo-controlled, double-blind, multicenter study. J Bone Miner Res. 2009 Apr;24(4):719-25.

32. McClung MR, Bolognese MA, Sedarati F, Recker RR, Miller PD. Efficacy and safety of monthly oral ibandronate in the prevention of postmenopausal bone loss. Bone. 2009 Mar;44(3):418-22.

33. McClung M, Miller P, Recknor C, Mesenbrink P, Bucci-Rechtweg C, Benhamou CL. Zoledronic acid for the prevention of bone loss in postmenopausal women with low bone mass: A randomized controlled trial. Obstet Gynecol. 2009 Nov;114(5):999-1007.

34. Matsumoto T, Hagino H, Shiraki M, Fukunaga M, Nakano T, Takaoka K, Morii H, Ohashi Y, Nakamura T. Effect of daily oral minodronate on vertebral fractures in japanese postmenopausal women with established osteoporosis: A randomized placebo-controlled double-blind study. Osteoporos Int. 2009 Aug;20(8):1429-37.

35. Yan Y, Wang W, Zhu H, Li M, Liu J, Luo B, Xie H, Zhang G, Li F. The efficacy and tolerability of once-weekly alendronate 70 mg on bone mineral density and bone turnover markers in postmenopausal chinese women with osteoporosis. J Bone Miner Metab. 2009;27(4):471-8.

36. Boonen S, Reginster JY, Kaufman JM, Lippuner K, Zanchetta J, Langdahl B, Rizzoli R, Lipschitz S, Dimai HP, Witvrouw R, Eriksen E, Brixen K, Russo L, Claessens F, Papanastasiou P, Antunez O, Su G, Bucci-Rechtweg C, Hruska J, Incera E, Vanderschueren D, Orwoll E. Fracture risk and zoledronic acid therapy in men with osteoporosis. N Engl J Med. 2012 Nov;367(18):1714-23.

37. Chapurlat RD, Laroche M, Thomas T, Rouanet S, Delmas PD, de Vernejoul MC. Effect of oral monthly ibandronate on bone microarchitecture in women with osteopenia-a randomized placebo-controlled trial. Osteoporos Int. 2013 Jan;24(1):311-20.

38. Yang Y, Luo X, Yan F, Jiang Z, Li Y, Fang C, Shen J. Effect of zoledronic acid on vertebral marrow adiposity in postmenopausal osteoporosis assessed by MR spectroscopy. Skeletal Radiol. 2015 Oct;44(10):1499-505.

39. Lewiecki EM, Keaveny TM, Kopperdahl DL, Genant HK, Engelke K, Fuerst T, Kivitz A, Davies RY, Fitzpatrick LA. Once-monthly oral ibandronate improves biomechanical determinants of bone strength in women with postmenopausal osteoporosis. J Clin Endocrinol Metab. 2009 Jan;94(1):171-80.

Supplemental Table 2: Characteristics of Included Studies in Individual Patient Level Data Meta-Analysis

| Study | Population | Treatment Allocation | BMD Characteristics | CKD Characteristics | Laboratory | Fracture Risk Factors |
| --- | --- | --- | --- | --- | --- | --- |
| 1^st^ author  Date of publication  Dates | Number included  Age  Sex  Race | Bisphosphonate  Placebo | Hip BMD (g/cm^2^)  Spine BMD (g/cm^2^) | eGFR (ml/min/1.73 m^2^)  CKD Stages | Urine albumin/creatinine (mg/g)  Urine protein/creatinine (mg/g)  Parathyroid hormone (pmol/L)  Serum calcium (mmol/L)  Phosphate (mmol/L)  Alkaline phosphatase (U/L)  Bone-specific alkaline phosphatase (U/L) | BMI  History of Fracture  Smoking  Family history of fracture  Corticosteroids  Rheumatoid arthritis |
| Riis(16)  (2001)  N/A | N =240  Mean Age = 66.7 (4.9)  Sex = 100% Female  Race = 100% White | Bisphosphonate = 159  Placebo = 81 | N/A | Mean eGFR = 74.0 (12.7)  CKD Stages =  41 (17.1%) Stage G1 CKD  164 (68.3%) Stage G2 CKD  34 (14.2%) Stage G3A CKD  1 (0.4%) Stage G3B CKD | N/A  N/A  4.3 (2.0)  N/A  N/A  154.2 (38.2)  52.1 (22.3) | 24.5 (3.6)  1 (0.4%)  77 (32.1%)  N/A  1 (0.4%)  2 (0.8%) |
| Stakkestad(22) (2003)  1998-2000 | N = 625  Mean Age = 54.8 (3.2)  Sex = 100% Female  Race = 99.8% White | Bisphosphonate = 469  Placebo = 156 | Mean Hip = 0.880 (0.11)  Mean Spine = 0.924 (0.10) | Mean eGFR = 94.5 (11.4)  CKD Stages =  449 (71.8%) Stage G1 CKD  171 (27.4%) Stage G2 CKD  5 (0.8%) Stage G3A CKD | N/A  N/A  N/A  1.15 (0.14)  2.35 (0.08)  71.2 (19.3)  162.0 (44.3)* | 26.5 (4.4)  233 (37.3%)  138 (21.8%)  N/A  3 (0.5%)  4 (0.6%) |
| Delmas(23)  (2004)  1996-2000  BONE  2004 | N = 2937  Mean Age = 68.7 (6.2)  Sex = 100% Female  Race = 98.4% White | Bisphosphonate = 1960  Placebo = 977 | Mean Hip = 0.743 (0.11)  Mean Spine = 0.770 (0.10) | Mean eGFR = 65.4 (12.6)  CKD Stages =  139 (4.7%) Stage G1 CKD  1820 (62.0%) Stage G2 CKD  864 (29.4%) Stage G3A CKD  107 (3.6%) Stage G3B CKD  7 (0.2%) Stage G4 CKD | N/A  N/A  4.5 (1.9)**  1.17 (0.15)  2.41 (0.10)**  93.1 (44.0)  43.3 (19.4) | 26.0 (4.1)  21 (0.7%)  485 (16.5%)  N/A  8 (0.3%)  32 (1.1%) |
| Adami(24)  (2004)  N/A | N = 520  Mean Age =65.7 (4.5)  Sex = 100% Female  Race = 99.8% White | Bisphosphonate = 392  Placebo = 128 | Mean Hip = 0.743 (0.10)  Mean Spine = 0.747 (0.08) | Mean eGFR = 85.6 (13.3)  CKD Stages =  286 (55.0%) Stage G1 CKD  198 (38.1%) Stage G2 CKD  33 (6.3%) Stage G3A CKD  2 (0.4%) Stage G3B CKD  1 (0.2%) Stage G4 CKD | 14.1 (24.7)***  61.3 (61.3)***  N/A  1.17 (0.18)  2.37 (0.09)***  81.2 (22.0)  N/A | 25.5 (4.0)  232 (44.6%)  101 (19.4%)  N/A  1 (0.2%)  3 (0.6%) |
| Recker(26)  (2004)  N/A | N = 2852  Mean Age = 67.0 (5.1)  Sex = 100% Female  Race = 98.6% White | Bisphosphonate = 1906  Placebo = 946 | N/A | Mean eGFR = 62.7 (11.5)  CKD Stages =  70 (2.5%) Stage G1 CKD  1582 (55.5%) Stage G2 CKD  1089 (38.2%) Stage G3A CKD  107 (3.8%) Stage G3B CKD  4 (0.1%) Stage G4 CKD | N/A  N/A  4.1 (1.8)****  1.19 (0.15)  2.41 (0.10)****  87.6 (44.7)  55.5 (26.2)**** | 25.1 (3.2)  19 (0.7%)  583 (20.4%)  N/A  0 (0%)  75 (2.6%) |
| McClung(32)  (2009) | N = 160  Mean Age = 53.5 (3.7)  Sex = 100% Female  Race = 95.6% White | Bisphosphonate = 77  Placebo = 83 | Mean Hip = 0.875 (0.09)  Mean Spine = 0.905 (0.04) | Mean eGFR = 87.7 (13.0)  CKD Stages =  74 (46.3%) Stage G1 CKD  82 (51.3%) Stage G2 CKD  4 (2.5%) Stage G3A CKD | N/A  N/A  N/A  1.25 (0.14)  2.43 (0.08)  N/A  N/A | 27.3 (5.6)  26 (16.3%)  13 (8.1%)  29 (18.1%)  5 (3.1%)  0 (0%) |
| Lewiecki(39) (2009) | N = 94  Mean Age = 64.0 (6.6)  Sex = 100% Female  Race = 88.3% White | Bisphosphonate = 47  Placebo = 47 | Mean Hip = 0.753 (0.11)  Mean Spine = 0.788 (0.09) | Mean eGFR = 73.3 (15.0)  CKD Stages =  16 (17.0%) Stage G1 CKD  61 (64.5%) Stage G2 CKD  14 (14.9%) Stage G3A CKD  3 (3.2%) Stage G3B CKD | N/A  N/A  N/A  1.21 (0.15)  2.36 (0.09)  89.0 (27.8)  N/A | 26.3 (4.5)  21 (22.3%)  11 (11.7%)  N/A  0 (0%)  N/A |

*209 missing Bone-specific alkaline phosphatase

**2802 missing PTH, 135 missing calcium, 258 missing Bone-specific alkaline phosphatase

*******2 missing Urine albumin/creatinine, 165 missing Urine protein/creatinine, 1 missing serum calcium, 2237 missing Bone-specific alkaline phosphatase

****2728 missing PTH, 183 missing serum calcium

Supplemental Table 3: Cox PH models for fracture (interactions only)

|  |  | **B** | **SE** | **P value** |
| --- | --- | --- | --- | --- |
| CKD model | Bisphosphonate * Stage G2 CKD | 0.08213 | 0.33326 | 0.8054 |
|  | Bisphosphonate * Stage G3A CKD | 0.09693 | 0.34561 | 0.7791 |
|  | Bisphosphonate * Stage G3B CKD | **1.60436** | **0.69273** | **0.0206** |
|  | Bisphosphonate * Stage G4 CKD | n/a | n/a | n/a |
|  |  | **B** | **SE** | **P value** |
| eGFR model | Bisphosphonate * eGFR | -0.00396 | 0.00576 | 0.4913 |

Note: CKD=chronic kidney disease,

eGFR=estimated glomerular filtration rate, SE=standard error

N=7428, 740 events

Supplemental Table 4: Cox PH models for hypocalcemia (Ca<2.00mmol/L)

(interactions only)

|  |  | **B** | **SE** | **P value** |
| --- | --- | --- | --- | --- |
| CKD model | Bisphosphonate * Stage G2 CKD | 0.03802 | 0.35590 | 0.9149 |
|  | Bisphosphonate * Stage G3A CKD | 0.47836 | 0.39954 | 0.2312 |
|  | Bisphosphonate * Stage G3B CKD | 0.04745 | 0.58977 | 0.9359 |
|  | Bisphosphonate * Stage G4 CKD | n/a | n/a | n/a |
|  |  | **B** | **SE** | **P value** |
| eGFR model | Bisphosphonate * eGFR | -0.00478 | 0.00777 | 0.5380 |

Note: CKD=chronic kidney disease,

eGFR=estimated glomerular filtration rate, SE=standard error

N=7428, 388 events

Supplemental Figure 1: Search strategy

**Ageline search**

| **#** | **Query** |
| --- | --- |
| S63 | S45 AND S53 AND S62 |
| S62 | (S54 OR S55 OR S56 OR S57 OR S58 OR S59 OR S60 OR S61) |
| S61 | TX ((tripl* or trebl* or singl* or doubl*) W0 (blind* or dumm* or mask*)) |
| S60 | TX (random* or sham or placebo*) |
| S59 | TX "single-blind" |
| S58 | TX "double-blind" |
| S57 | TX "random allocation" |
| S56 | TX randomization |
| S55 | TX "randomized controlled" W0 trial* |
| S54 | DE "Randomized Controlled Trials" |
| S53 | (S46 OR S47 OR S48 OR S49 OR S50 OR S51 OR S52) |
| S52 | TX bmd |
| S51 | TX ((bone W3 (mass or mineral or density)) |
| S50 | TX "bone density" |
| S49 | TX fracture* |
| S48 | DE "Fractures" |
| S47 | TX osteoporosis |
| S46 | DE "Osteoporosis" |
| S45 | (S1 OR S2 OR S3 OR S4 OR S5 OR S6 OR S7 OR S8 OR S9 OR S10 OR S11 OR S12 OR S13 OR S14 OR S15 OR S16 OR S17 OR S18 OR S19 OR S20 OR S21 OR S22 OR S23 OR S24 OR S25 OR S26 OR S27 OR S28 OR S29 OR S30 OR S31 OR S32 OR S33 OR S34 OR S35 OR S36 OR S37 OR S38 OR S39 OR S40 OR S41 OR S42 OR S43 OR S44) |
| S44 | TX binosto |
| S43 | TX fosavance |
| S42 | TX fosamax |
| S41 | TX "alendronic acid" |
| S40 | TX alendronate |
| S39 | TX clasteon |
| S38 | TX ostac |
| S37 | TX loron |
| S36 | TX bonefos |
| S35 | TX "clodronic acid" |
| S34 | TX clodronate |
| S33 | TX didronel |
| S32 | TX didrocal |
| S31 | TX "mylan eti cal carepac" |
| S30 | TX etidrocol |
| S29 | TX "etidronic acid" |
| S28 | TX etidronate |
| S27 | TX hospira |
| S26 | TX aredia |
| S25 | TX pamidronate |
| S24 | TX olpadronate |
| S23 | TX "olpadronic acid" |
| S22 | TX cimadronate |
| S21 | TX "bone density conservation" W0 agent* |
| S20 | TX aclasta |
| S19 | TX zometa |
| S18 | TX diphosphonate* |
| S17 | TX reclast |
| S16 | TX "zoledronic acid" |
| S15 | TX zoledronate |
| S14 | TX skelid |
| S13 | TX "tiludronic acid" |
| S12 | TX tiludronate |
| S11 | TX boniva |
| S10 | TX bondronat |
| S9 | TX "ibandronic acid" |
| S8 | TX ibandronate |
| S7 | TX atelvia |
| S6 | TX actonel |
| S5 | TX "risedronic acid" |
| S4 | TX risedronate |
| S3 | TX "neridronic acid" |
| S2 | TX neridronate |
| S1 | TX bisphosphonate* |

**CINAHL search**

| **#** | **Query** |
| --- | --- |
| S59 | S44 AND S50 AND S57 |
| S58 | S44 AND S50 AND S57 |
| S57 | S51 OR S52 OR S53 OR S54 OR S55 OR S56 |
| S56 | TX randomiz* |
| S55 | TX "single-blind" |
| S54 | TX "double-blind" |
| S53 | TX "random allocation" |
| S52 | TX "randomized controlled" W0 trial* |
| S51 | DE "Randomized Controlled Trials" |
| S50 | S45 OR S46 OR S47 OR S48 OR S49 |
| S49 | TX ((bone W1 (mass OR mineral OR density)) |
| S48 | TX fracture OR TX fractures |
| S47 | DE "Fractures" |
| S46 | TX osteoporosis |
| S45 | DE "Osteoporosis" |
| S44 | S1 OR S2 OR S3 OR S4 OR S5 OR S6 OR S7 OR S8 OR S9 OR S10 OR S11 OR S12 OR S13 OR S14 OR S15 OR S16 OR S17 OR S18 OR S19 OR S20 OR S21 OR S22 OR S23 OR S24 OR S25 OR S26 OR S27 OR S28 OR S29 OR S30 OR S31 OR S32 OR S33 OR S34 OR S35 OR S36 OR S37 OR S38 OR S39 OR S40 OR S41 OR S42 OR S43 |
| S43 | TX binosto |
| S42 | TX fosavance |
| S41 | TX fosamax |
| S40 | TX "alendronic acid" |
| S39 | TX alendronate |
| S38 | TX clasteon |
| S37 | TX ostac |
| S36 | TX loron |
| S35 | TX bonefos |
| S34 | TX "clodronic acid" |
| S33 | TX clodronate |
| S32 | TX didronel |
| S31 | TX didrocal |
| S30 | TX "mylan eti cal carepac" |
| S29 | TX etidrocol |
| S28 | TX "etidronic acid" |
| S27 | TX etidronate |
| S26 | TX hospira |
| S25 | TX aredia |
| S24 | TX pamidronate |
| S23 | TX olpadronate |
| S22 | TX "olpadronic acid" |
| S21 | TX cimadronate |
| S20 | TX aclasta |
| S19 | TX zometa |
| S18 | TX diphosphonate* |
| S17 | TX reclast |
| S16 | TX "zoledronic acid" |
| S15 | TX zoledronate |
| S14 | TX skelid |
| S13 | TX "tiludronic acid" |
| S12 | TX tiludronate |
| S11 | TX boniva |
| S10 | TX bondronat |
| S9 | TX "ibandronic acid" |
| S8 | TX ibandronate |
| S7 | TX atelvia |
| S6 | TX actonel |
| S5 | TX "risedronic acid" |
| S4 | TX risedronate |
| S3 | TX "neridronic acid" |
| S2 | TX neridronate |
| S1 | TX bisphosphonate* |

**Cochrane search**

ID     Search

#1     bisphosphonate?:ti,ab,kw

#2     neridronate:ti,ab,kw

#3     "neridronic acid":ti,ab,kw

#4     risedronate:ti,ab,kw

#5     "risedronic acid":ti,ab,kw

#6     actonel:ti,ab,kw

#7     atelvia:ti,ab,kw

#8     ibandronate:ti,ab,kw

#9     "ibandronic acid":ti,ab,kw

#10     bondronat:ti,ab,kw

#11     boniva:ti,ab,kw

#12     tiludronate:ti,ab,kw

#13     "tiludronic acid":ti,ab,kw

#14     skelid:ti,ab,kw

#15     zoledronate:ti,ab,kw

#16     "zoledronic acid":ti,ab,kw

#17     reclast:ti,ab,kw

#18     aclasta:ti,ab,kw

#19     zometa:ti,ab,kw

#20     MeSH descriptor: [Diphosphonates] this term only

#21     MeSH descriptor: [Alendronate] this term only

#22     MeSH descriptor: [Clodronic Acid] this term only

#23     MeSH descriptor: [Etidronic Acid] this term only

#24     cimadronate:ti,ab,kw

#25     "olpadronic acid":ti,ab,kw

#26     olpadronate:ti,ab,kw

#27     pamidronate:ti,ab,kw

#28     aredia:ti,ab,kw

#29     hospira:ti,ab,kw

#30     diphosphonate?:ti,ab,kw

#31     etidronate:ti,ab,kw

#32     "etidronic acid":ti,ab,kw

#33     etidrocol:ti,ab,kw

#34     "mylan eti cal carepac":ti,ab,kw

#35     didrocal:ti,ab,kw

#36     didronel:ti,ab,kw

#37     clodronate:ti,ab,kw

#38     "clodronic acid":ti,ab,kw

#39     bonefos:ti,ab,kw

#40     loron:ti,ab,kw

#41     ostac:ti,ab,kw

#42     clasteon:ti,ab,kw

#43     alendronate:ti,ab,kw

#44     "alendronic acid":ti,ab,kw

#45     fosamax:ti,ab,kw

#46     fosavance:ti,ab,kw

#47     binosto:ti,ab,kw

#48     {or #1-#47}

#49     MeSH descriptor: [Osteoporosis] this term only

#50     osteoporosis:ti,ab,kw

#51     MeSH descriptor: [Fractures, Bone] this term only

#52     fracture?:ti,ab,kw

#53     MeSH descriptor: [Bone Density] this term only

#54     (bone near/1 (mass or mineral or density)):ti,ab,kw

#55     43-54

#56     MeSH descriptor: [Randomized Controlled Trials as Topic] explode all trees

#57     MeSH descriptor: [Randomized Controlled Trial] this term only

#58     MeSH descriptor: [Random Allocation] this term only

#59     MeSH descriptor: [Double-Blind Method] this term only

#60     MeSH descriptor: [Single-Blind Method] this term only

#61     MeSH descriptor: [Placebos] this term only

#62     (random* or sham or placebo*):ti,ab,kw

#63     ((singl* or doubl*) next (blind* or dumm* or mask*)):ti,ab,kw

#64     ((tripl* or trebl*) next (blind* or dumm* or mask*)):ti,ab,kw

#65     randomized controlled trial:pt

#66     pragmatic clinical trial:pt

#67     {or #56-#66}

#68     #48 and #55 and #67

#69     MeSH descriptor: [Animals] explode all trees

#70     MeSH descriptor: [Humans] explode all trees

#71     #69 not (#69 and #70)

#72     #68 not #71

#73     (#48 and #55 and #67) not #71

**EMBASE Search**

1. bisphosphonate?.mp.

2. neridronate.mp.

3. neridronic acid.mp.

4. risedronate.mp.

5. risedronic.mp.

6. actonel.mp.

7. atelvia.mp.

8. ibandronate.mp.

9. ibandronic acid.mp.

10. bondronat.mp.

11. boniva.mp.

12. tiludronate.mp.

13. tiludronic acid.mp.

14. skelid.mp.

15. zoledronate.mp.

16. zoledronic acid.mp.

17. reclast.mp.

18. aclasta.mp.

19. zometa.mp.

20. bisphosphonic acid derivative/ or alendronic acid/ or alendronic acid plus colecalciferol/ or calcium carbonate plus colecalciferol plus risedronic acid/ or calcium carbonate plus risedronic acid/ or clodronic acid/ or etidronic acid/ or ibandronic acid/ or incadronic acid/ or minodronic acid/ or neridronic acid/ or olpadronic acid/ or pamidronic acid/ or risedronic acid/ or tiludronic acid/ or zoledronic acid/

21. minodronic acid.mp.

22. incadronic acid.mp.

23. cimadronate.mp.

24. olpadronic acid.mp.

25. olpadronate.mp.

26. pamidronate.mp.

27. aredia.mp.

28. hospira.mp.

29. diphosphonate?.mp.

30. etidronate.mp.

31. etidonic acid.mp.

32. etidrocol.mp.

33. mylan eti cal carepac.mp.

34. didrocal.mp.

35. didronel.mp.

36. clodronate.mp.

37. clodronic acid.mp.

38. bonefos.mp.

39. loron.mp.

40. ostac.mp.

41. clasteon.mp.

42. alendronate.mp.

43. alendronic acid.mp.

44. fosamax.mp.

45. fosavance.mp.

46. binosto.mp.

47. or/1-46

48. osteoporosis/ or idiopathic osteoporosis/ or involutional osteoporosis/ or postmenopause osteoporosis/ or primary osteoporosis/ or senile osteoporosis/

49. osteoporosis.ti,ab.

50. fracture/

51. fracture?.ti,ab.

52. bone density/

53. (bone adj1 (mass or mineral or density)).mp.

54. or/48-53

55. Randomized Controlled Trial.pt.

56. Pragmatic Clinical Trial.pt.

57. exp Randomized Controlled Trials as Topic/

58. "Randomized Controlled Trial (topic)"/

59. Randomized Controlled Trial/

60. Randomization/

61. Random Allocation/

62. Double-Blind Method/

63. Double-Blind Procedure/

64. Double-Blind Studies/

65. Single-Blind Method/

66. Single-Blind Procedure/

67. Single-Blind Studies/

68. Placebos/

69. Placebo/

70. (random* or sham or placebo*).ti,ab,hw.

71. ((singl* or doubl*) adj (blind* or dumm* or mask*)).ti,ab,hw.

72. ((tripl* or trebl*) adj (blind* or dumm* or mask*)).ti,ab,hw.

73. or/55-72

74. 47 and 54 and 73

75. animals/ not (animals/ and human$.mp.)

76. animal$/ not (animal$/ and human$/)

77. or/75-76

78. 74 not 77

79. limit 78 to embase

80. limit 78 to english language

**MEDLINE Search**

1. bisphosphonate?.mp.

2. neridronate.mp.

3. neridronic acid.mp.

4. risedronate.mp.

5. risedronic acid.mp.

6. actonel.mp.

7. atelvia.mp.

8. ibandronate.mp.

9. ibandronic acid.mp.

10. bondronat.mp.

11. boniva.mp.

12. tiludronate.mp.

13. tiludronic acid.mp.

14. skelid.mp.

15. zoledronate.mp.

16. zoledronic acid.mp.

17. reclast.mp.

18. aclasta.mp.

19. zometa.mp.

20. diphosphonates/ or alendronate/ or clodronic acid/ or etidronic acid/

21. incadronic acid.mp.

22. minodronic acid.mp.

23. cimadronate.mp.

24. olpadronic acid.mp.

25. olpadronate.mp.

26. pamidronate.mp.

27. aredia.mp.

28. hospira.mp.

29. diphosphonate?.mp.

30. etidronate.mp.

31. etidronic acid.mp.

32. etidrocol.mp.

33. mylan eti cal carepac.mp.

34. didrocal.mp.

35. didronel.mp.

36. clodronate.mp.

37. clodronic acid.mp.

38. bonefos.mp.

39. loron.mp.

40. ostac.mp.

41. clasteon.mp.

42. alendronate.mp.

43. alendronic acid.mp.

44. fosamax.mp.

45. fosavance.mp.

46. binosto.mp.

47. or/1-46

48. Osteoporosis/

49. osteoporosis.ti,ab.

50. Fractures, Bone/

51. fracture?.ti,ab.

52. Bone Density/

53. (bone adj1 (mass or mineral or density)).mp.

54. or/48-53

55. Randomized Controlled Trial.pt.

56. Pragmatic Clinical Trial.pt.

57. exp Randomized Controlled Trials as Topic/

58. "Randomized Controlled Trial (topic)"/

59. Randomized Controlled Trial/

60. Randomization/

61. Random Allocation/

62. Double-Blind Method/

63. Double Blind Procedure/

64. Double-Blind Studies/

65. Single-Blind Method/

66. Single-Blind Procedure/

67. Single-Blind Studies/

68. Placebos/

69. Placebo/

70. (random* or sham or placebo*).ti,ab,hw.

71. ((singl* or doubl*) adj (blind* or dumm* or mask*)).ti,ab,hw.

72. ((tripl* or trebl*) adj (blind* or dumm* or mask*)).ti,ab,hw.

73. or/55-72

74. animals/ not (animals/ and human$.mp.)

75. animal$/ not (animal$/ and human$/)

76. or/74-75

77. 47 and 54 and 73

78. 77 not 76

79. limit 78 to english language

80. remove duplicates from 79

81. limit 80 to medline

Supplemental Figure 2: Risk of Bias Assessment


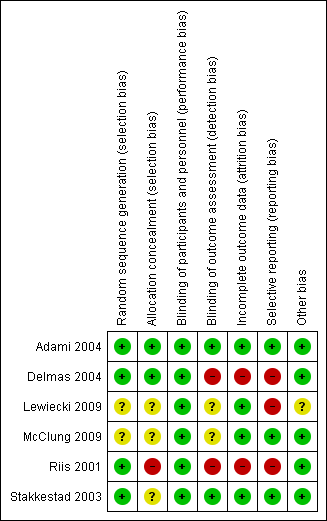

Supplement: sj-docx-1-cjk-10.1177_20543581241283523 – Supplemental material for The Efficacy and Safety of Bisphosphonate Therapy for Osteopenia/Osteoporosis in Patients With Chronic Kidney Disease: A Systematic Review and Individual Patient-Level Meta-Analysis of Placebo-Controlled Randomized Trials [file sj-docx-1-cjk-10.1177_20543581241283523.docx]
